# Large-Scale Cerebellar-Cerebral Network Alterations Underlying Impaired Social Predictive Processing in Cerebellar Neurodegeneration

**DOI:** 10.1007/s12311-026-02095-y

**Published:** 2026-10-06

**Authors:** Libera Siciliano, Nicole Urbini, Giusy Olivito, Michelle Pisu, Martina Tanieli, Andrea Ciricugno, Viola Oldrati, Federico Giove, Renato Borgatti, Zaira Cattaneo, Cosimo Urgesi, Maria Leggio

**Affiliations:** 1 Department of Human Sciences, Link Campus University, Rome, Italy; 2 Ataxia Research Laboratory, LIFE - Institute for Research and Health Care - Santa Lucia IRCCS, Rome, Italy; 3 Department of Psychology, Sapienza University of Rome, Rome, Italy; 4 IRCCS Mondino Foundation, Pavia, Italy; 5 Scientific Institute, IRCCS E. Medea, Bosisio Parini, LC Italy; 6 Neuroimaging Laboratory, LIFE - Institute for Research and Health Care - Santa Lucia IRCCS, Rome, Italy; 7 Centro Ricerche Enrico Fermi, Rome, Italy; 8 Department of Brain and Behavioral Sciences, University of Pavia, Pavia, Italy; 9 Department of Human and Social Sciences, University of Bergamo, Bergamo, Italy; 10 Department of Human and Social Sciences, Universitas Mercatorum, Rome, Italy

**Keywords:** Predictive learning models, Social interaction, Functional magnetic resonance imaging, Cerebellar diseases, Basal ganglia

## Abstract

The cerebellum is a key node for detecting and adjusting social-prediction internal models. Yet social prediction has been scantly investigated in neurodegenerative cerebellar patients (CB). Thus, we examined CB social-prediction abilities and related cerebellar-cerebral networks, using tailored tasks and resting-state fMRI. Thirteen adult CB and twenty-six matched controls completed three computerized prediction tasks, where contextual priors activate specific internal models setting expectations which may influence recognition of ambiguous targets (emotion expressions, personality-traits, non-social objects). Accuracy and response times were analysed through 2 × 2 × 2 mixed-effects ANOVAs. A congruency index (CI) reflecting whether response was driven by contextual influence was derived from performance efficiency scores and analysed through a 2 × 2 mixed-effects ANOVA. Participants underwent 3T brain MRI, including T1-weighted and resting-state fMRI sequences. Posterior and vermian cerebellar lobules were chosen *a-priori* as regions of interest and used in a Seed-Based fMRI analysis examining cerebellar-cerebral functional connectivity (FC). FC values from patients’ altered connectivity clusters were extracted and correlated with CIs. CB performed worse in the personality-traits task, showing higher reliance on contextual cues. Cerebellar-cerebral underconnectivity was found within networks involved in mentalizing and learning. In CB, higher CI in the personality-traits task, reflecting greater contextual influence and failure to revise behavioural responses, significantly correlated with right lobule VIII-right putamen underconnectivity. These findings indicate reduced flexibility in updating higher-order social predictions when contextual expectations conflict with incoming information, accompanied by altered cerebellar-cerebral FC. Exploratory findings further point to cerebellar-basal ganglia connectivity as a potentially relevant network component of predictive social cognition, warranting further investigation.

**Supplementary Information:**

The online version contains supplementary material available at https://doi.org/10.1007/s12311-026-02095-y.

## Introduction

 Social cognition refers to the set of cognitive and affective processes that enable individuals to understand, interpret, and respond appropriately to others’ behaviours, emotions, and intentions, thereby supporting effective social interactions [1, 2]. Navigating the social world requires not only the online processing of social cues, but also the ability to anticipate others’ behaviours, emotional reactions, and intentions in dynamically changing environments [3–5]. Such predictions rely on previously acquired knowledge about the social environment and on the contextual framework in which social interactions occur. Indeed, social events are inherently context-dependent, and the same action, behaviour, or emotional expression may convey different meanings depending on the embedding context [6, 7].

Current accounts of social cognition increasingly conceptualize these processes within predictive coding frameworks [8–10]. According to these models, the brain continuously generates predictions about incoming sensory information and compares them with actual inputs. Sensory signals related to actions, emotional states, or social events are interpreted in light of prior expectations (“priors”), which are shaped by previously experienced statistical regularities of the environment [11]. When incoming information is novel or deviates from prior expectations, prediction errors are generated and internal models are updated accordingly in order to optimize future predictions [10]. Within social interactions, contextual information represents one of the primary sources of priors [12, 13]. Contextual priors have been shown to influence multiple social prediction processes, including action prediction and recognition [14] and the recognition of emotional facial expressions [15–17]. Specifically, congruent contextual information facilitates recognition of actions, objects, or emotional expressions, whereas incongruent contexts impair performance [14, 18]. The congruency effect therefore represents an important index of the extent to which contextual information is used predictively to guide social perception and behaviour.

Traditionally, social cognition and social prediction processes have been attributed to the activation of distributed cerebral networks encompassing the Default Mode Network (DMN) and its mentalizing-related components, including the temporoparietal junction (TPJ), dorsomedial and ventromedial prefrontal cortex (dmPFC, vmPFC), precuneus, inferior and middle temporal gyrus, and posterior cingulate cortex, as well as other social regions, such as the inferior and superior frontal gyrus [19, 20]. Nevertheless, in the last decades, the cerebellum has emerged as a key structure supporting not only motor but also cognitive, affective, and social processes [21–27]. Within the predictive coding framework, the cerebellum is widely believed to contribute to predictive processing by comparing incoming stimuli with expected patterns, thereby generating internal models that anticipate future events and detect mismatches between predicted and actual outcomes [28–30].

A complementary account of cerebellar predictive functioning is provided by the sequence detection theory proposed by Leggio and colleagues [31, 32]. According to this framework, the cerebellum detects and simulates temporally and spatially organized sequences of events. By processing incoming information in relation to previously learned patterns, as well as novel information for which no specific expectation has yet been established, the cerebellum contributes to the generation and refinement of internal models capable of anticipating future events and signalling mismatches between predicted and actual outcomes [28, 31, 32]. Importantly, sequence detection is conceived as a domain-general process that may support different functional domains through cerebello-cerebral interactions, extending beyond motor behaviour to cognitive and social processes [31–33]. Converging evidence suggests that the cerebellum contributes to social behaviour by supporting the generation and updating of predictions about others’ actions, emotions, and intentions [34–36]. From this perspective, social interactions provide a rich source of sequential information and regularities that can be learned and incorporated into cerebellar internal models. Indeed, recent evidence indicates that the cerebellum is involved in learning and representing dynamic social action sequences, supporting the acquisition of regularities that can subsequently guide social predictions [37, 38]. Once acquired, internal models may support different aspects of predictive social processing, including the identification and anticipation of ongoing or novel sequences of others’ actions, as well as the detection of events that violate previously established expectations [38]. In the latter case, predictions may rely on contextual priors derived from the social environment, allowing the individual to evaluate whether an observed action, behaviour, or emotional expression is consistent with what is expected in a given context [8–10]. Thus, while predictive processing may involve the anticipation of how social events unfold based on learned sequential regularities, it may also support the detection of violations when incoming information is incongruent with contextual expectations.

This view is supported by the extensive functional connectivity (FC) between posterior cerebellar regions and cortical areas involved in social cognition, including core nodes of the mentalizing components of DMN [22, 24, 39–41]. Through these cerebello-cortical loops, the cerebellum is thought to contribute to the construction of internal models that integrate contextual information, detect prediction errors, and facilitate adaptive responses during social interactions [28, 34, 42]. In particular, posterior cerebellar regions such as Crus I, Crus II, and lobule IX have been consistently implicated in the processing of social sequences and predictive social information [35, 42, 43].

Further support for this framework comes from clinical studies of patients with cerebellar degeneration or focal cerebellar lesions. Damage involving posterior cerebellar regions, particularly Crus I and Crus II, is associated with impairments in multiple domains of social cognition, including emotion recognition, intention understanding, and social inference [34, 35, 44–47]. Moreover, patients with cerebellar degeneration have shown impaired performance in reconstructing the correct order of socially meaningful action sequences, further supporting the involvement of the cerebellum in social sequencing processes [48]. Importantly, recent evidence revealed deficits specifically involving tasks that require the use of contextual information and the prediction of others’ mental or behavioural states, suggesting a disruption of predictive mechanisms normally supported by the cerebellum [34, 49]. Consistent with this interpretation, cerebellar patients show altered FC between posterior cerebellar regions and cortical areas involved in social cognition, indicating that impaired cerebello-cortical communication may underlie deficits in adaptive social prediction [34, 41, 50, 51].

Despite this growing body of evidence, the neural basis of context-based social prediction deficits in cerebellar patients – and the specific role of cerebello-cortical FC in supporting contextual predictive processing – remain poorly understood. Disruption of cerebellar-cortical FC in patients with cerebellar degeneration likely underlies their impaired ability to anticipate social outcomes and detect social prediction errors [34, 50], but direct correlational evidence linking specific patterns of reduced connectivity to performance at ecologically valid and context-based social prediction tasks is still lacking.

The present study addresses this gap by investigating the role of the cerebellar-cerebral networks in context-based social prediction in a group of individuals with cerebellar degenerative pathology compared to age- and education-matched healthy subjects (HS). Participants completed a battery of computerized context-based prediction tasks specifically designed to evaluate the use of contextual priors – either congruent or incongruent with the target stimulus at varying levels of ambiguity – across two social domains (emotion expression prediction and personality traits prediction) and a non-social control domain (non-social object prediction). The experimental paradigm was adapted from Ferrari et al. [52] and allows dissociation of the effect of contextual congruency from that of stimulus ambiguity, enabling a fine-grained characterization of predictive processing. In addition, resting-state FC analyses were conducted to examine whether disrupted cerebello-cortical connectivity between posterior cerebellar regions and cortical areas implicated in social cognition could underlie observed behavioural deficits.

Based on the existing literature, we hypothesized that patients with cerebellar degeneration will show deficits relative to healthy controls in social prediction tasks, with particular difficulty in conditions presenting contextual cues incongruent with the target stimulus – reflecting impaired prediction-error updating. We further expected these deficits to be most pronounced for the highest-order social prediction condition, namely personality traits inference, which places greater demands on the construction of complex social internal models. Finally, we hypothesized that these behavioural deficits will be associated with reduced FC between posterior cerebellar regions and cortical areas involved in social cognition and predictive processing, including frontal, temporal, and parietal regions of the mentalizing components of DMN.

## Materials and Methods

### Participants

Thirteen CB patients were enrolled (mean age ± SD = 49.54 ± 12.88; mean educational level ± SD = 15.08 ± 2.66; M/F = 5/8; Raven’s 47 Progressive Matrices test (CPM-47) [53] mean correct score ± SD = 29.89 ± 3.15, expressed as the total number of years of formal education completed; mean disease duration expressed in years = 14.46 ± 9.22). The inclusion criteria were: (i) presence of diffuse cerebellar atrophy; (ii) right-handedness (assessed using the Edinburgh Handedness Inventory – EHI [54]); (iii) eligibility for MRI scanning; (iv) confirmed diagnosis of a cerebellar neurodegenerative disorder; (v) general intellectual functioning within the normal range (assessed using the CPM-47 [53]); (vi) age between 18 and 75 years; (vii) symptom duration exceeding six months. Exclusion criteria included: (i) diagnosis of additional neurological or psychiatric disorders; (ii) evidence of cortical lesions identified through MRI inspection by an expert radiologist. Clinical and demographic characteristics of each patient are presented in Table 1.

**Table 1:** Demographic and clinical characteristics of CB patients

ID	Age	M/F	Education (years)	Diagnosis	Disease duration (years)	Number of repeats	CPM-47	ICARS
CB1	54	M	13	SCA2	9	38 ± 1	29.8	38
CB2	34	F	13	SCA28	20	n.a.	31.0	11
CB3	58	M	13	SPG7	14	n.a.	31.3	30
CB4	56	F	17	Episodic Ataxia	35	n.a.	29.3	14
CB5	51	F	18	SCA28	26	n.a.	31.6	32
CB6	33	F	16	SCA2	5*	40	30.4	20
CB7	73	M	17	SCA2	12	35 ± 1	27.4	36
CB8	64	F	18	SCA2	21	37 ± 1	28.7	50
CB9	36	F	18	SCA1	5	51 ± 1	27.7	41
CB10	52	F	13	SCA2	12	n.a.	35.0	54
CB11	51	M	10	SCA2	11	39 ± 1	33.3	41
CB12	30	M	13	SCA1	2*	50 ± 1	31.0	23
CB13	52	F	17	Friedreich’s Ataxia	16	n.a.	22.1	45

*F* female, *M* male, *CPM-47* correct scores for Raven’s 47 Progressive Matrices test [53], *ICARS* International Cooperative Ataxia Rating Scale, *SCA* Spinocerebellar Ataxia, *SPG* Spastic paraplegia; ICARS range: minimum score 0 (absence of motor deficits), maximum score 100 (maximum presence of motor deficits) [55]. Disease duration refers to the time interval (in years) from symptom onset to the date of assessment. When information on symptom onset was unavailable, disease duration was calculated from the date of genetic diagnosis (cases marked with *). The number of expanded genetic repeats is not reported for all patients, as not all conditions have a genetic basis or, in some cases, the number of expanded repeats was not specified at the time of genetic diagnosis

Additionally, twenty-six HS were recruited (mean age ± SD = 50.35 ± 5.06; mean educational level ± SD = 15.62 ± 2.26, expressed as the total number of years of formal education completed; M/F = 10/16; CPM-47 [53] mean correct score ± SD = 32.14 ± 1.77). The inclusion criteria were: (i) age of at least 40 years, to match the mean age of the CB group; (ii) right-handedness (assessed using the EHI [54]); (iii) eligibility for the MRI protocol; (iv) general intellectual functioning within the normal range (assessed using the CPM-47 [53]). Exclusion criteria included: (i) diagnosis of neurological and/or psychiatric disorders; (ii) cardiovascular disease; (iii) history of head trauma, infarction, aneurysm, stroke, or ischemia.

All participants underwent the social prediction tests and a 3T brain MRI scanning. CB patients underwent a neurological evaluation and the International Co-operative Ataxia Rating Scale (ICARS) [55] was administered to quantify the severity of cerebellar motor deficits (Mean score ± DS = 33.46 ± 13.41). Conventional MRI scans, acquired as part of this research study, were inspected by an expert medical radiologist to confirm the presence of cerebellar atrophy (see Fig. S1 in Supplementary Materials to inspect cerebellar T1-weighted images of CB patients). For the HS group, conventional MRI was used to exclude the presence of brain/cerebellar abnormalities.

No significant differences were found between the two groups in mean age (t = -0.282, *p* = 0.779) or educational level (t = -0.661, *p* = 0.513), as evidenced by the t-test analyses. Similarly, no difference was observed in gender distribution (χ² = 0.00, *p* = 1.000), as assessed using the chi-squared (χ²) test. The present study was approved by the Scientific Ethics Committee of IRCCS Santa Lucia Foundation (n. CE/2024_026) in accordance with the principles expressed in the Declaration of Helsinki. Furthermore, each participant reviewed and signed informed consent for the processing of sensitive data.

### Behavioural Tasks

Participants completed three computerized context-based prediction tasks, designed to evaluate predictive processes based on contextual information. Two tasks addressed the social domain, each focusing on different aspects (emotion expression or personality traits prediction), whereas the third assessed prediction during the perception of non-social stimuli, serving as a control task. The experimental paradigm was adapted from a previous study [52]. All tasks were programmed in E-Prime (Psychology Software Tools, Inc., Pittsburgh, PA, USA), shared an identical trial structure, and were administered on a laptop computer in a single experimental session, in counterbalanced order.

At the beginning of each task, participants read the instructions presented on the screen. Each trial started with a fixation cross displayed for a variable interval (1000–1500 ms), separating consecutive trials. This was followed by the presentation of a contextual visual cue (priming stimulus, presented for 1000 ms), intended to induce expectations about the subsequent ambiguous target stimulus. Immediately after the prime, a target stimulus appeared for 500 ms. The relationship between the contextual prime and the target could be either congruent (e.g., a prime with positive valence followed by a positive facial expression) or incongruent (e.g., a negative prime followed by a positive expression). Participants were required to categorize the target using a two-alternative forced-choice procedure by selecting the label that best described the stimulus (e.g., “joy” when the target displayed a positive emotional expression or “fear” when it conveyed a negative emotion).

In addition, targets were presented at two levels of ambiguity (low vs. high). This manipulation allowed us to examine whether participants’ responses varied as a function of the amount of information conveyed by the target stimulus. For instance, when the target was highly ambiguous, participants might rely more strongly on the contextual information provided by the prime. The two response labels were displayed in the lower left and lower right portions of the screen during target presentation and remained visible until a response was given, with no time constraints. Participants responded using two keyboard keys (Z and M), corresponding respectively to the left and right labels. The spatial position of the labels was counterbalanced across participants.

**Fig. 1:**
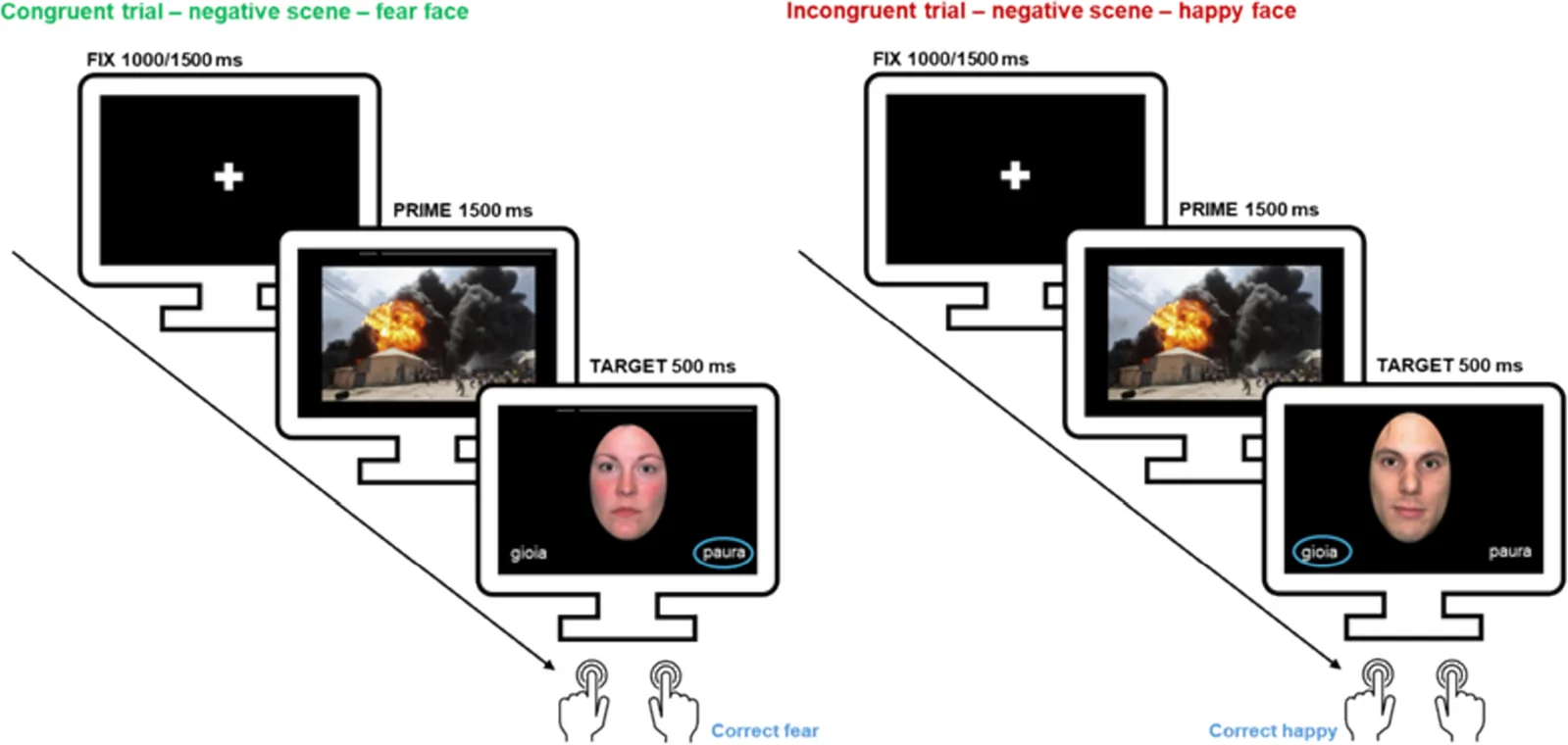
Emotion expression prediction task’s examples of congruent trials (left) and incongruent trials (right). Emotion labels in Italian (gioia/joy and paura/fear) are displayed in the lower part of the screen

**Fig. 2:**
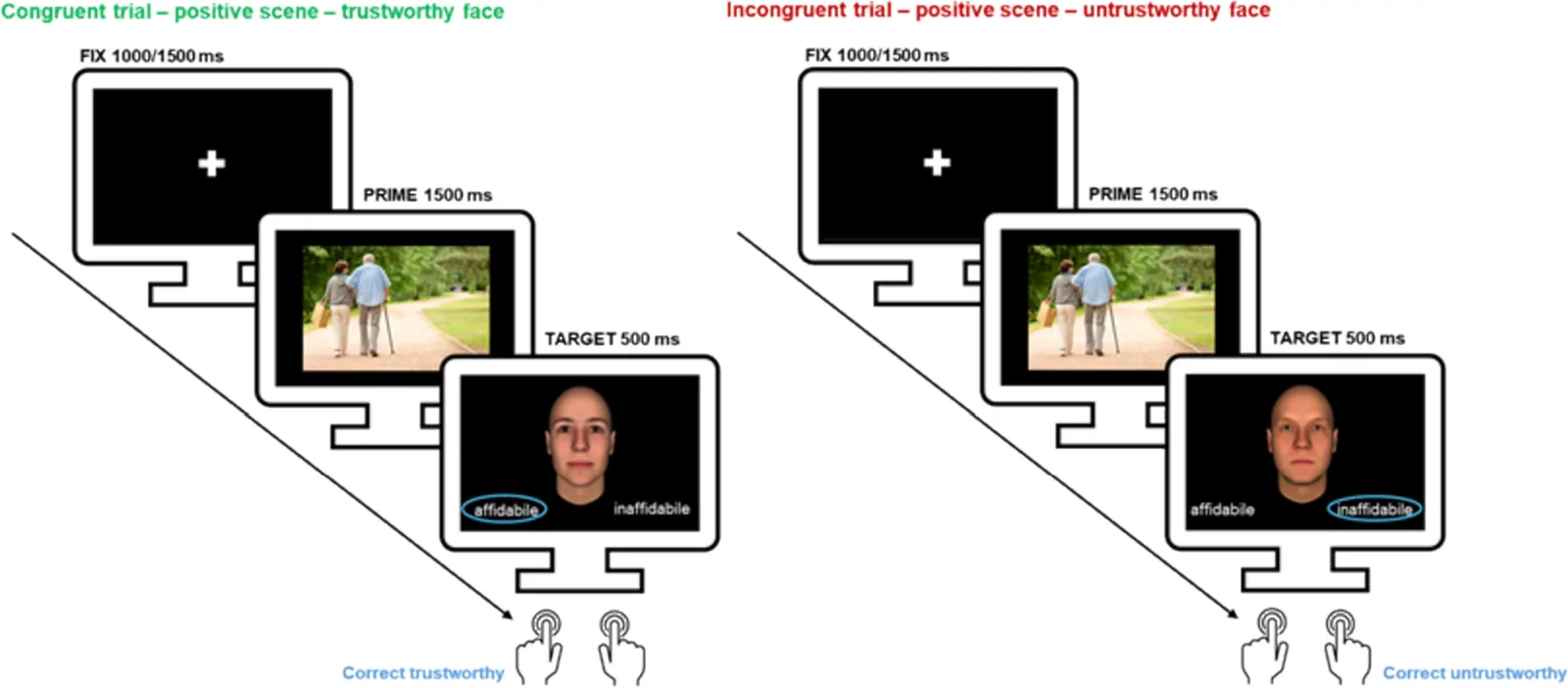
Personality traits prediction task’s examples of congruent trials (left) and incongruent trials (right). Personality traits labels in Italian (affidabile/trustworthy and inaffidabile/untrustworthy) are displayed in the lower part of the screen

**Fig. 3:**
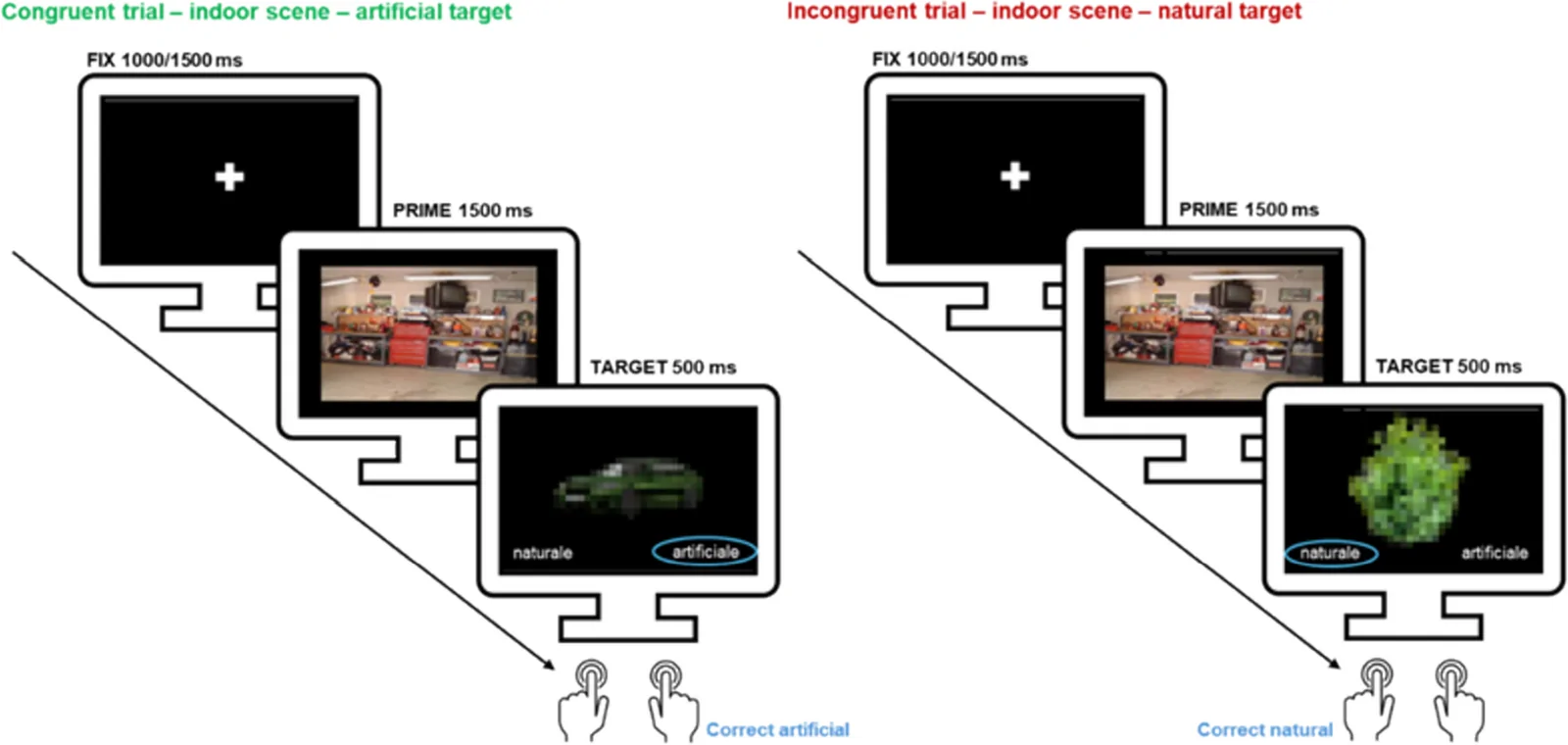
Non-social prediction task’s examples of congruent trials (left) and incongruent trials (right). Object category labels in Italian (naturale/natural and artificiale/artificial) are displayed in the lower part of the screen

Each task included 160 trials, consisting of 20 trials for each of the eight possible combinations generated by crossing target category (e.g., joy vs. fear), congruency with the prime (congruent vs. incongruent), and ambiguity level (low vs. high). Performance was evaluated in terms of accuracy (ACC) and response times (RTs) for correct responses, expressed in milliseconds. The specific features of each paradigm are described below (see Figs. 1, 2 and 3 for examples of trial sequences).

#### Task 1. Emotion Expressions Prediction Task

The target stimuli represented images of male and female faces displaying either positive (joy) or negative (fear) emotional expressions, drawn from the NimStim Face Stimulus Set developed by Tottenham and colleagues [56]. Emotional facial expressions were manipulated to vary in ambiguity by applying two levels of facial morphing (20% and 30%). Faces with 20% morphing (e.g., 20% joy blended with 80% neutral) represented higher level of ambiguity, whereas faces with 30% morphing (e.g., 30% joy and 70% neutral) represented lower level of ambiguity. Participants were required to categorize each target face in a forced-choice task by selecting the label that best described the expression (joy or fear). Each target face was preceded by a contextual prime consisting of photographs depicting everyday situations capable of eliciting either positive (e.g., a birthday celebration) or negative emotional reactions (e.g., a theft). The emotional valence of the contextual prime could either match the emotional expression of the subsequent face (congruent condition; e.g., a negative situation followed by a fear expression; Fig. 1, left) or mismatch it (incongruent condition; e.g., a negative situation followed by a joyful expression; Fig. 1, right).

#### Task 2. Personality Traits Prediction Task

The target stimuli were computer-generated male and female faces conveying different degrees of trustworthiness (trustworthy vs. untrustworthy). These faces were drawn from a validated database developed by Oosterhof and Todorov [57]. Trustworthiness was manipulated by varying facial features along a parametric continuum ranging from − 2 to + 2 standard deviations, producing four levels (− 2 very untrustworthy, − 1 untrustworthy, + 1 trustworthy, + 2 very trustworthy). This manipulation allowed the creation of two levels of ambiguity in the target stimuli. Participants were instructed to categorize each face using a forced-choice procedure by selecting the label that best described the stimulus (trustworthy or untrustworthy). Before the presentation of the target face, a contextual image was shown as a prime. These primes consisted of photographs depicting real-life situations illustrating behaviours with either positive valence (e.g., volunteering) or negative valence (e.g., acts of physical aggression). Depending on the relationship between the prime and the target, trials could be congruent (e.g., a positive valence scene followed by a trustworthy face; Fig. 2, left) or incongruent (e.g., a positive valence scene followed by an untrustworthy face; Fig. 2, right).

#### Task 3. Non-social Prediction Task

In this task, the targets were degraded images depicting either natural objects (e.g., a bush) or artificial objects (e.g., a car). The stimuli were selected from an established database developed by Konkle and Oliva [58]. To manipulate perceptual uncertainty, the images were blurred at two different pixel resolutions, creating two levels of ambiguity. Objects blurred at 15 mm pixels constituted the high ambiguity condition, whereas those blurred at 11 mm pixels corresponded to the low ambiguity condition. Participants were required to identify the category of the target object through a two-alternative forced-choice decision, indicating whether the stimulus represented a “natural” or an “artificial” item. Each object image was preceded by a contextual prime intended to activate expectations about the upcoming target. These primes consisted of photographs portraying either outdoor environments (e.g., a countryside landscape) or indoor environments (e.g., a garage). The relationship between the contextual scene and the target object varied across trials: in congruent trials, the contextual prime matched the object category (e.g., an indoor scenario followed by an artificial object; Fig. 3, left), whereas in incongruent trials the context did not correspond to the object category (e.g., an indoor scenario followed by a natural object; Fig. 3, right).

### MRI Acquisition Protocol

MRI data were acquired on a 3T Siemens MAGNETOM Prisma scanner using a 64-channel head-and-neck coil. Anatomical data included T1-weighted (T1w) images acquired via a fat-suppressed MEMPRAGE sequence (TR = 2500 ms, TEs = 1.67,3.48,5.29,7.10 ms, TI = 1080 ms, flip angle = 8∘, GRAPPA = 2,1mm3 isotropic voxels, FoV = 256 × 256 mm2, 176 slices; combined online via RMS) and T2-weighted (T2w) images collected using a SPACE sequence (TR/TE = 3200/564 ms, GRAPPA = 2,1mm3 isotropic voxels, FoV = 256 × 240 mm2, 176 slices). Both anatomical sequences incorporated volumetric navigators for prospective motion correction [59].

For functional MRI (fMRI), resting-state data were collected using a multi-echo T2*-weighted GE-EPI sequence (TR = 980 ms, TEs = 12.80,30.42,48.02 ms, flip angle = 63°, Multiband factor = 4, GRAPPA = 2, 2.8 mm3 isotropic voxels, FoV = 208 × 208 mm2, 52 slices, 488 volumes) [60, 61]. Two spin-echo EPI scans with reversed phase-encoding directions (TE/TR = 80/7000 ms) were acquired for susceptibility distortion correction. During fMRI acquisition, participants were instructed to remain at rest, avoid thinking about anything in particular, and fixate on a white cross displayed against a black background. The stimulus was projected onto a translucent screen inside the scanner bore and viewed through a mirror attached to the head coil.

### Resting‑State fMRI Data Pre‑processing

Data were organized according to BIDS standard [62] using dcm2bids (v3.1.1) and preprocessed through an integrated workflow combining fMRIPrep (v25.1.4) and tedana (v25.0.1 [63]). Anatomical T1w images were corrected for intensity non-uniformity using N4, skull-stripped with ANTs, and segmented into tissue classes using FSL FAST, while cortical surfaces were reconstructed with FreeSurfer (recon-all) incorporating T2w images for refined border identification. T1w images were subsequently non-linearly normalized to MNI152NLin2009cAsym space. For functional data, the first 3 volumes were discarded (resulting in 7 total dummy scans including automated scanner procedures, leaving 485 volumes for analysis). Functional runs were realigned for head motion using FSL MCFLIRT and corrected for susceptibility distortions via FSL TOPUP using the reversed phase-encoded spin-echo images. BOLD runs were co-registered to the anatomical T1w reference via boundary-based registration (bbregister). Following motion and distortion correction in native space, multi-echo time series were processed with tedana for adaptive masking, monoexponential T2*/S0 fitting, and T2*-weighted optimal combination [64]. Dimensionality reduction via PCA and decomposition via RobustICA were performed to classify components into BOLD and non-BOLD noise according to the decision-tree framework [65]. Denoised time series were then spatially transformed into MNI space in a single interpolation step. Finally, using custom MATLAB (R2025b) and AFNI (3dRSFC) scripts, data were spatially smoothed using an 8 mm FWHM Gaussian kernel, band-pass filtered (0.008–0.09 Hz), and regressed for residual head-motion parameters and physiological confounds extracted via aCompCor.

Detailed descriptions of MRI preprocessing (fMRIPrep) and multi-echo denoising (tedana) are provided in the Supplementary Materials.

### Definition of Regions of Interest and Seed‑Based Analyses

Regions of interest (ROIs) were defined according to anatomical cerebellar lobular parcellation provided by the Automated Anatomical Labeling 3 (AAL3) atlas [66] and transformed into EPI native geometry. Residual time series extracted from the unsmoothed preprocessed data were averaged within 28 AAL3-derived ROIs. From this anatomical parcellation, a subset of posterior hemispheric and vermian cerebellar lobules was selected a priori as seeds for the subsequent seed-to-voxel FC analyses (i.e. posterior and vermian lobules Crus I and II, VIIb, VIII and IX). The selection was intended to encompass cerebellar regions implicated not only in social and emotional processing, but also in higher-order cognitive processes relevant to prediction, including the integration and updating of internal representations [39, 42, 50, 67–71]. Specifically, while Crus I, Crus II, and lobule IX have been consistently implicated in social cognition [39, 42], lobule VIIb has been implicated in social processing [72] as well as in higher-order executive functions relevant to predictive updating [69]. Although evidence for a specific social role of lobule VIII is less established, its recruitment has been reported during self-guided sequence-based navigation involving increased planning and prediction demands [71]. Thus, the inclusion of lobules VIIb and VIII was intended to provide broader coverage of posterior cerebellar regions potentially contributing to the predictive and updating processes engaged by the tasks employed in this study, beyond regions with a more consistently established role in social cognition. Seed-to-voxel FC analyses were performed on the smoothed MNI-normalized datasets. Individual correlation maps were Fisher-*z* transformed prior to second-level modeling in SPM12 (Statistical Parametric Mapping, SPM12; Wellcome Centre for Human Neuroimaging, London, UK; https://www.fil.ion.ucl.ac.uk/spm/*).*

At the second level, a two-sample t test was used to explore differences in FC in the identified ROIs between CB and HS groups. To account for inter-individual variations in brain morphology, total grey matter volume (GMV) was extracted for each participant by integrating the tissue probability maps derived from the anatomical T1w segmentation (FSL FAST/FreeSurfer) in standard space. Individual total GMV values were subsequently included as a nuisance covariate in the second-level statistical analyses. The cerebellum was excluded from the analysis by entering an implicit inclusion mask that included only the cerebral cortex. The results were considered significant at p values < 0.05 after FWE cluster-level correction (clusters formed with *p* < 0.001 at the uncorrected level).

### Statistical Analyses

#### Sample Size Estimation

Given the rarity of cerebellar disorders, the available sample was limited. Thus, a sensitivity power analysis was conducted using G*Power 3.1 [73] with the “as in SPSS” option to estimate the minimum detectable effect size with the obtained sample (*N* = 39). Considering a 2 × 2 × 2 mixed repeated-measures ANOVA (two groups × two levels of congruency × two levels of ambiguity), with α = 0.05 and a target power of 0.80, the analysis indicated a minimum detectable effect size of f = 0.319, reflecting a medium effect size according to conventional benchmarks [74].

#### Data Handling

For each participant and for each context-based prediction task, two performance indices were computed: accuracy, calculated as the proportion of correct responses (range: 0–1), with each response coded as 1 for correct and 0 for incorrect, and reaction times (RTs) for correct responses, expressed in milliseconds.

Prior to statistical analyses, data screening was performed at both the trial and participant levels. First, data were cleaned at the trial level separately for each participant, task and each of the four experimental conditions resulting from the combination of congruency (congruent vs. incongruent) and ambiguity level (high vs. low). Trials with RTs exceeding ± 3 standard deviations from the participant’s condition-specific mean RT were identified and removed to minimize the influence of unusually fast and slow responses, as they were considered anticipatory or excessively delayed responses. Subsequently, participant-level outlier screening was performed separately for each task and behavioural measure on the aggregated accuracy and RT measures using boxplots in JASP. Values falling below Q1 − 1.5 × IQR or above Q3 + 1.5 × IQR were identified as outliers. Outlier exclusion was measure-specific: participants identified as outliers for a given measure within a task were excluded only from the corresponding analysis, while their data were retained for other measures for which they did not meet the outlier criterion. This approach avoided the unnecessary exclusion of otherwise valid behavioural data across measures. Following this data-screening procedure, a cleaned dataset was obtained for each task. The resulting samples are reported in Table S1 in the Supplementary Materials, which includes information on sample size and demographic characteristics. To verify the comparability of the groups after data cleaning, independent-samples t-tests were conducted on age and years of education, while differences in gender distribution were assessed using chi-square (χ²) tests. Results are reported in Table S1.

A mixed-effects ANOVA was conducted to compare Accuracies and mean RTs between CB and HS groups. For each task, a 2 × 2 × 2 mixed ANOVA was performed with target ambiguity (high vs. low) and prime–target congruency (congruent vs. incongruent) as within-subject factors, and group (HS vs. CB) as a between-subject factor. Statistical significance was set at *p* ≤ 0.05 for all analyses.

To obtain an integrative estimate of contextual priors influence on behavioural performance, while simultaneously reducing the number of dependent variables, an additional index was calculated.

First, Accuracy and RTs were combined into a single efficiency index, the Rate-Correct Score (RCS), following previous work [75]. This metric reflects the number of correct responses produced per unit of time, thus capturing the trade-off between speed and accuracy. The RCS was computed according to the formula “RCS = (C / ΣRT) × 1000”, where C represents the number of correct responses and ΣRT is the sum of RTs across trials. The multiplication by 1000 converts milliseconds into seconds. Within the present tasks, higher RCS values indicate greater overall efficiency, corresponding to faster and more accurate performance. Prior to subsequent analyses, participant-level outlier screening was also performed on RCS values using the same boxplot-based criterion described above. Participants identified as outliers were excluded from the corresponding RCS-based analyses. Group comparisons on age and years of education, after outlier removal, were performed using independent-samples t-tests, while gender distributions were compared using chi-square (χ²) tests. The results of these comparisons are reported in Table S1 of the Supplementary Materials. On the basis of this metric, a Congruency Index (CI) was then calculated to quantify the impact of contextual priors on performance. The CI was computed using the formula “(Congruent Trials RCS – Incongruent Trials RCS) / [(Congruent Trials RCS + Incongruent Trials RCS) / 2]”. This computation involves two steps: first, the difference between congruent and incongruent conditions is calculated; second, this difference is divided by their average (i.e., the mean of congruent and incongruent RCS values). In this way, the CI represents a normalized measure of the relative performance advantage associated with congruent contextual information and can be interpreted as an index of contextual facilitation. Positive values indicate better performance in congruent relative to incongruent trials, suggesting that participants effectively used contextual cues to guide their responses. Values close to zero reflect minimal differences between conditions, whereas negative values would indicate relatively better performance in incongruent trials. Importantly, by scaling the difference by the average performance across conditions, the CI controls for individual variability in overall efficiency, providing a standardized measure of the influence of context in prediction. Both RCS and CI were computed separately for each task and for each level of target ambiguity, thereby preserving the experimental manipulations while substantially reducing the number of variables.

The CIs for each context-based prediction task were inserted into a 2 × 2 mixed-design ANOVA with ambiguity level (high vs. low) as a within-subject factor and group (HS vs. CB) as a between-subject factors, to further confirm precedent mixed ANOVAs results by providing a direct and more specific test of the congruency effect. Statistical significance was set at *p* ≤ 0.05.

For each ANOVAs, significant interactions were followed up with Holm-Bonferroni–corrected post-hoc [76]. ANOVA effect sizes were reported as partial eta-squared (η²p) and interpreted using conventional thresholds: 0.01 = small, 0.06 = medium, 0.14 = large.

Following these analyses, only the CIs derived from tasks showing a significant between-group difference were entered into an exploratory brain–behaviour correlation analysis. The CI was selected because it captures the overall behavioural effect of contextual priors while reducing the number of separate brain–behaviour correlations that would have been required if accuracy and RTs measures had been analysed independently. Specifically, we investigated whether patterns of altered cerebellar-cerebral FC in CB patients were associated with the use of contextual priors during predictive processing. To this end, individual FC estimates (beta values) extracted from the clusters exhibiting significantly altered connectivity in the patient group were correlated, within the CB group, with the corresponding CI values using Spearman’s rank-order correlations. Results were considered significant at *p* ≤ 0.05, and Benjamini–Hochberg correction was applied to account for multiple comparisons [77]. Since the CI integrates both response ACC and RTs and given the presence of motor impairment in the patient group, additional sensitivity analyses were planned to assess whether significant brain–behaviour associations were attributable to overall motor severity assessed by ICARS. For associations warranting this control, partial correlations were computed between FC patterns and CIs while controlling for the total motor severity score.

Statistical analyses were conducted using the statistical software program JASP 0.95.3.0 (JASP Team, 2024).

## Results

### Context-Based Prediction Tasks

As participant-level outlier exclusion was performed separately for each task and behavioural measure, final sample sizes differed slightly across Accuracy, RT, and CIs analyses. No significant difference was found in age, education, and gender distribution between CB and HS in any of the resulting samples. Sample sizes and the corresponding demographic comparisons for each analysis are reported in Table S1 in Supplementary Materials. Descriptive statistics for each mixed-effects ANOVAs are presented in the Supplementary materials (Tables S2–S8). 

As reported in the Methods, the sensitivity power analysis indicated a minimum detectable effect size of f = 0.319 (η²p ≈ .092). All significant main effects and interactions of interest exceeded this threshold. Conversely, effects smaller than this threshold cannot be excluded when interpreting non-significant findings.

#### Accuracy and RTs Results

**Fig. 4:**
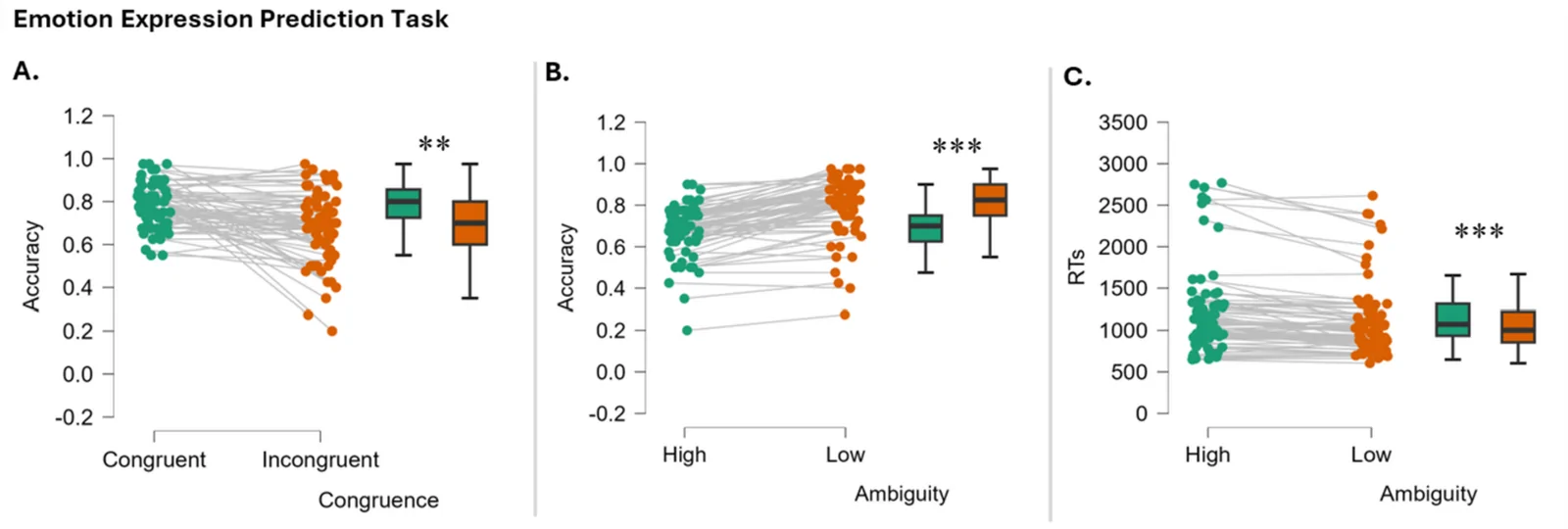
Emotion expressions prediction task significant main effect. **A**. Mean accuracy of congruent vs. incongruent trials. (**B**) Mean accuracy of high ambiguity vs. low ambiguity trials. (**C**) Mean RTs of high ambiguity vs. low ambiguity trials. In the boxplots, hinges of the box represent the first and the third quartile, with the line in the box representing the median, the bottom and top whiskers representing the smallest and the largest value no further than 1.5*IQR from the lower and the upper hinges, respectively; the dots represent a single participant’s value. Significant difference = *p < 0.05; **p < 0.01; ***p < 0.001

Regarding the Emotion expression prediction task, 2 × 2 × 2 mixed-effects ANOVA for accuracy revealed a significant main effect of Congruency [F (1, 32) = 9.868; p = 0.004; η²p = 0.236], explained by lower accuracy in trials where the target was incongruent with the prime (M = 0.684; SEM = 0.019) compared to trials where the target was congruent with the prime (M = 0.785; SEM = 0.012) (Fig. 4A). Additionally, the analysis showed a significant main effect of Ambiguity [F (1, 32) = 117.700; *p* < 0.001; η²p = 0.786] driven by lower accuracy in trials with more ambiguous targets (M = 0.676; SEM = 0.014) compared to those with less ambiguous targets (M = 0.793; SEM = 0.017) (Fig. 4B). Regarding RTs analysis, a 2 × 2 × 2 mixed-effects ANOVA revealed a significant main effect of Ambiguity [F (1, 34) = 20.670; p < 0.001; η²p = 0.378)], explained by participants’ faster RTs in trials where the target was less ambiguous (M = 1123.16; SEM = 52.035) compared to trials where the target was more ambiguous (M = 1234.86; SEM = 62.46) (Fig. 4C). No other significant interaction was found either for accuracy or RTs (all *p* > 0.05). Descriptive statistics are reported in Table S2.

**Fig. 5:**
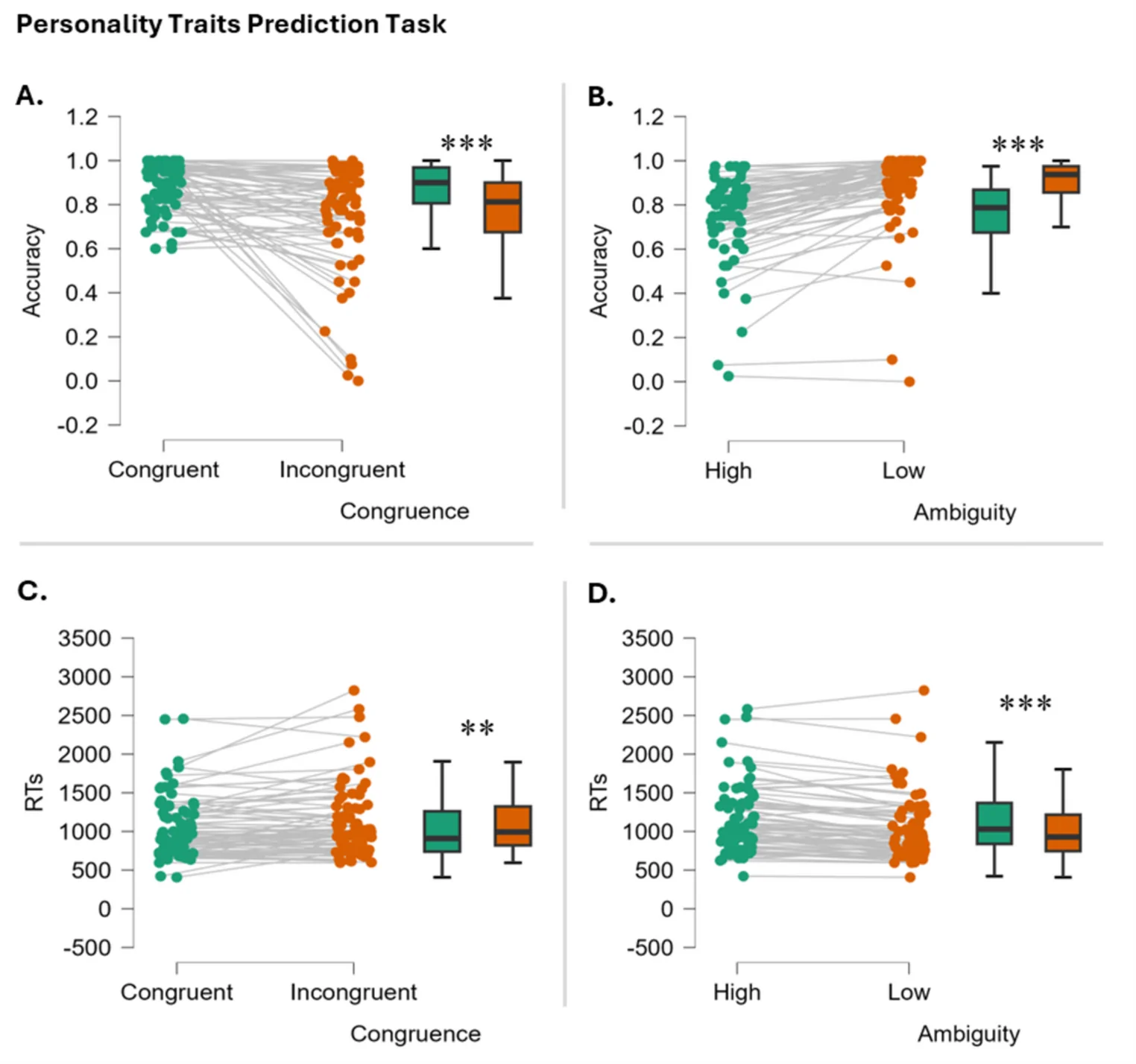
Personality traits prediction task significant main effects. **A**. Mean accuracy of congruent vs. incongruent trials. (**B**) Mean accuracy of high ambiguity vs. low ambiguity trials. (**C**) Mean RTs of congruent vs. incongruent trials. (**D**) Mean RTs of high ambiguity vs. low ambiguity trials. In the boxplots, hinges of the box represent the first and the third quartile, with the line in the box representing the median, the bottom and top whiskers representing the smallest and the largest value no further than 1.5*IQR from the lower and the upper hinges, respectively; the dots represent a single participant’s value. Significant difference = *p < 0.05; **p < 0.01; ***p < 0.001

Regarding the Personality traits prediction task, the 2 × 2 × 2 mixed-effects ANOVA for accuracy showed a significant main effect of Congruency [F (1, 33) = 17.824; *p* < 0.001; η²p = 0.351] explained by lower accuracy in trials where the target was incongruent with the prime (M = 0.743; SEM = 0.028) compared to trials where the target was congruent with the prime (M = 0.874; SEM = 0.013) (Fig. 5A). Descriptive statistics are reported in Table S2.

**Fig. 6:**
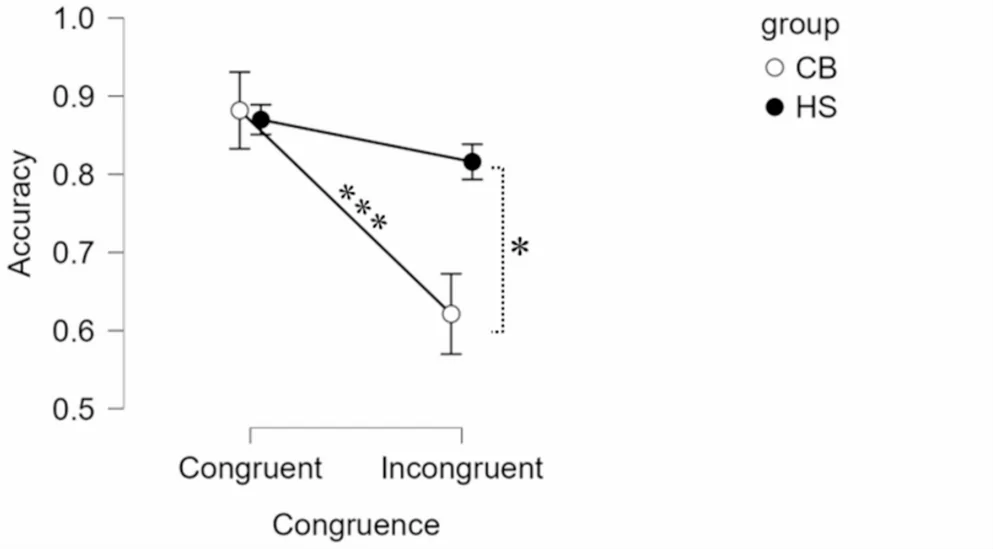
Personality traits prediction task significant interaction effect. Between and within groups differences as revealed by pairwise comparisons of the congruency x group interaction effect. Data points represent group average accuracy; error bars represent SEM. Significant difference = *p<0.05; **p<0.01; ***p<0.001

A significant Congruency x Group interaction was also found [(F (1, 33) = 7.689; *p* = 0.009; η²p = 0.189)]. Holm-Bonferroni corrected post-hoc highlighted that CB group was significantly less accurate in recognizing the correct personality trait in trials where the target was incongruent with the prime (M = 0.621; SEM = 0.06) compared to trials where the target was congruent with the prime (M = 0.881; SEM = 0.019) (pcorr < 0.001, d = 1.59, mean difference = 0.261, 95% Confidence Interval from mean difference [0.095, 0.426]) (Fig. 6). Conversely, no significant difference was detected in HS between congruent and incongruent trials (pcorr = 0.566, d = 0.329, mean difference = 0.054, 95% Confidence Interval from mean difference [-0.073, 0.181]) (Fig. 6). Moreover, CB patients were significantly less accurate in recognizing the correct personality trait in incongruent trials compared to HS subjects (M = 0.815; SEM = 0.022) (pcorr = 0.04, d = -1.189, mean difference = -0.195, 95% Confidence Interval from mean difference [-0.395, 0.005]), while no difference was found between CB and HS in congruent trials (pcorr = 0.683, d = 0.072, mean difference = 0.012, 95% Confidence Interval from mean difference [-0.069, 0.093]) (Fig. 6). Descriptive statistics are reported in Table S3.

The accuracy analysis also showed a significant main effect of Ambiguity [F (1, 33) = 94.627; *p* < 0.001; η²p = 0.741] explained by lower accuracy in trials in which the target was more ambiguous (M = 0.743; SEM = 0.022) compared to trials in which the target was less ambiguous (M = 0.874; SEM = 0.021) (Fig. 5B). Descriptive statistics are reported in Table S2.

Concerning the RTs 2 x 2 x 2 mixed-effects ANOVA, the analysis showed a significant main effect of Congruency [F (1, 35) = 8.197; p = 0.007; η²p = 0.190)], explained by faster RTs for all participants in trials where the target was congruent with the prime (M = 1048.29; SEM = 48.006) compared to RTs in incongruent trials (M = 1139.4; SEM = 55.495) (Fig. 5C). Additionally, a significant main effect of Ambiguity was found [F (1, 35) = 41.557; p < 0.001; η²p = 0.543)], explained by participants’ faster RTs in trials where the target was less ambiguous (M = 1037.85; SEM = 50.931) compared to trials where the target was more ambiguous (M = 1149.84; SEM = 52.547) (Fig. 5D). Descriptive statistics are reported in Table S2. 

No other significant interaction was found either for accuracy or RTs (all *p* > 0.05).

**Fig. 7:**
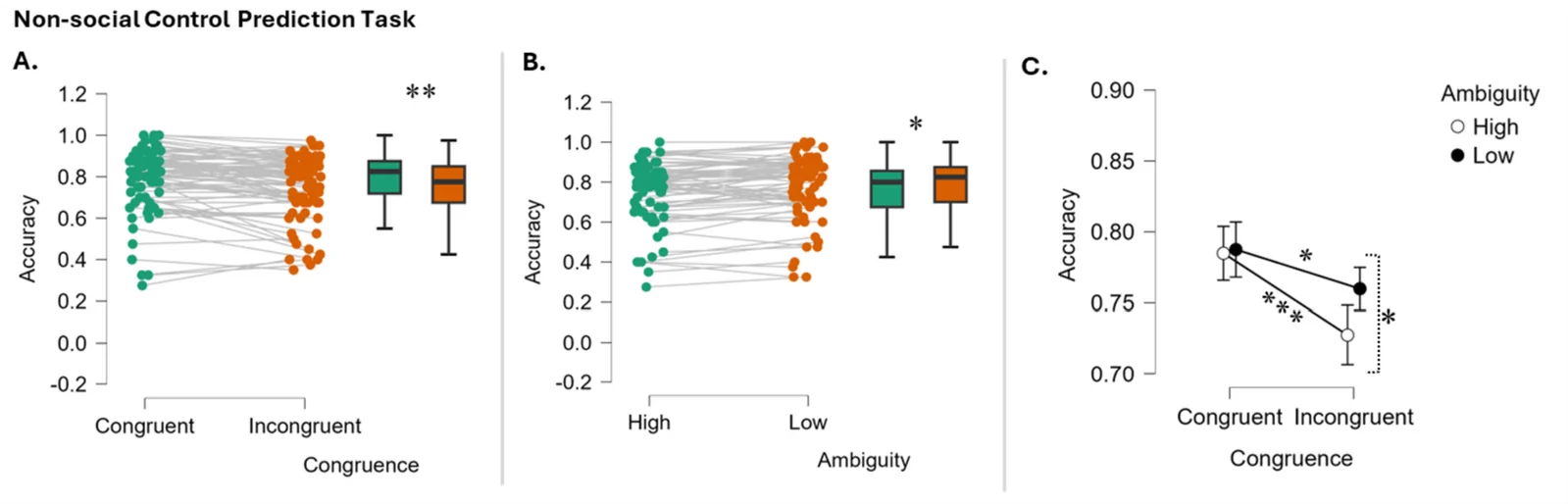
Non-social control prediction task significant main effect. **A**. Mean accuracy of Congruent vs. Incongruent trials. (**B**) Mean accuracy of high ambiguity vs. low ambiguity trials. In the boxplots, hinges of the box represent the first and the third quartile, with the line in the box representing the median, the bottom and top whiskers representing the smallest and the largest value no further than 1.5*IQR from the lower and the upper hinges, respectively; the dots represent a single participant’s value. (**C**) Within-subjects differences as revealed by pairwise comparisons of the congruency x ambiguity interaction effect. Data points represent group average accuracy; error bars represent SEM. Significant difference = *p < 0.05; **p < 0.01; ***p < 0.001

Concerning the Non-social control prediction task, a 2 x 2 x 2 mixed-effects ANOVA for accuracy showed a significant main effect of Congruency [F (1, 36) = 16.163; *p* < 0.001; η²p = 0.310] explained by lower accuracy in trials where the target was incongruent with the prime (M = 0.743; SEM = 0.017) compared to trials where the target was congruent with the prime (M = 0.786; SEM = 0.017) (Fig. 7A). The analysis also revealed a significant main effect of Ambiguity [F (1, 36) = 7.173; *p* = 0.011; η²p = 0.166] explained by lower accuracy in trials in which the target was more ambiguous (M = 0.756; SEM = 0.018) compared to trials in which the target was less ambiguous (M = 0.773; SEM = 0.017) (Fig. 7B). Descriptive statistics are reported in Table S2.

A significant Congruency x Ambiguity interaction was also found [(F (1, 36) = 4.261; *p* = 0.046; η²p = 0.106)]. Holm-Bonferroni corrected post-hoc highlighted that all participants were significantly less accurate in recognizing objects in trials where the target was incongruent with the prime compared to congruent ones, both when the target was more ambiguous (pcorr < 0.001, d = 0.453, mean difference = 0.065, 95% CI from mean difference [0.021, 0.109]) (Incongruent: M = 0.727; SEM = 0.026; Congruent: M = 0.784; SEM = 0.024) and less ambiguous (pcorr = 0.043, d = 0.236, mean difference = 0.034, 95% CI from mean difference [-0.003, 0.071]) (Incongruent: M = 0.759; SEM = 0.024; Congruent: M = 0.787; SEM = 0.026) (Fig. 7C). Moreover, participants were significantly less accurate in incongruent trials when the targets were more ambiguous compared to when they were less ambiguous (pcorr = 0.003, d = -0.257, mean difference = -0.037, 95% Confidence Interval from mean difference [-0.065, -0.009]) (Fig. 7C). Descriptive statistics are reported in Table S6.

No other significant main effect or interaction was found for accuracy neither for RTs (all *p* > 0.05).

#### RCS Congruency Indices Results

**Fig. 8:**
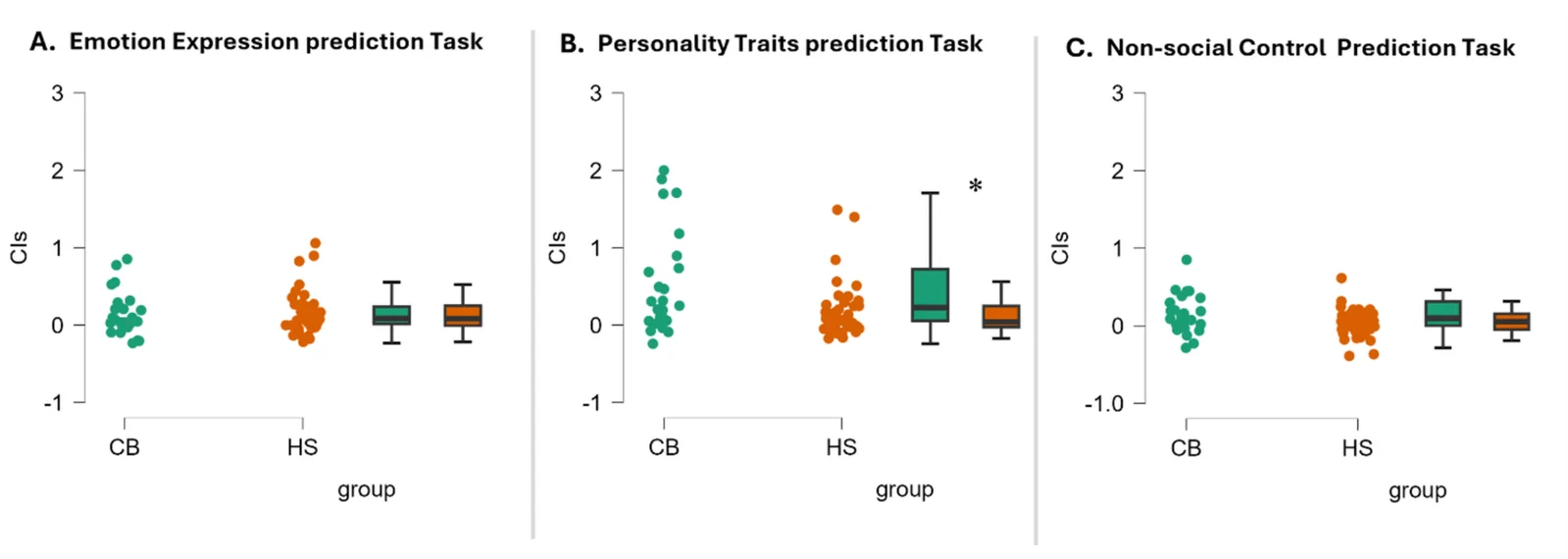
CIs in each context-based prediction task and for each group. Between groups differences as revealed by pairwise comparisons of the Group main effect. Data points represent group mean CIs; error bars represent SEM. Significant difference = *p < 0.05; **p < 0.01; ***p < 0.001

Regarding the Emotion expression and Non-social control prediction tasks, the two 2 x 2 mixed-effects ANOVAs for CIs revealed no significant effect or interaction (all *p* > 0.05) (Fig. 8A and C, respectively). Conversely, in Personality traits prediction task, a 2 x 2 mixed-effects ANOVA for CIs revealed a significant main effect of Group [F(1, 35) = 4.657; *p* = 0.038; η²p = 0.117], explained by higher CI in patients (M = 0.505; SEM = 0.129) compared to controls (M = 0.159; SEM = 0.048) (Fig. 8B), which reflected greater contextual influence and failure to revise responses based on prediction-error updating, thus matching accuracy results and further highlighting patients’ dependency on contextual cues in mentalizing. Descriptive statistics are reported in Table S8.

### Seed-Based FC Results

**Fig. 9:**
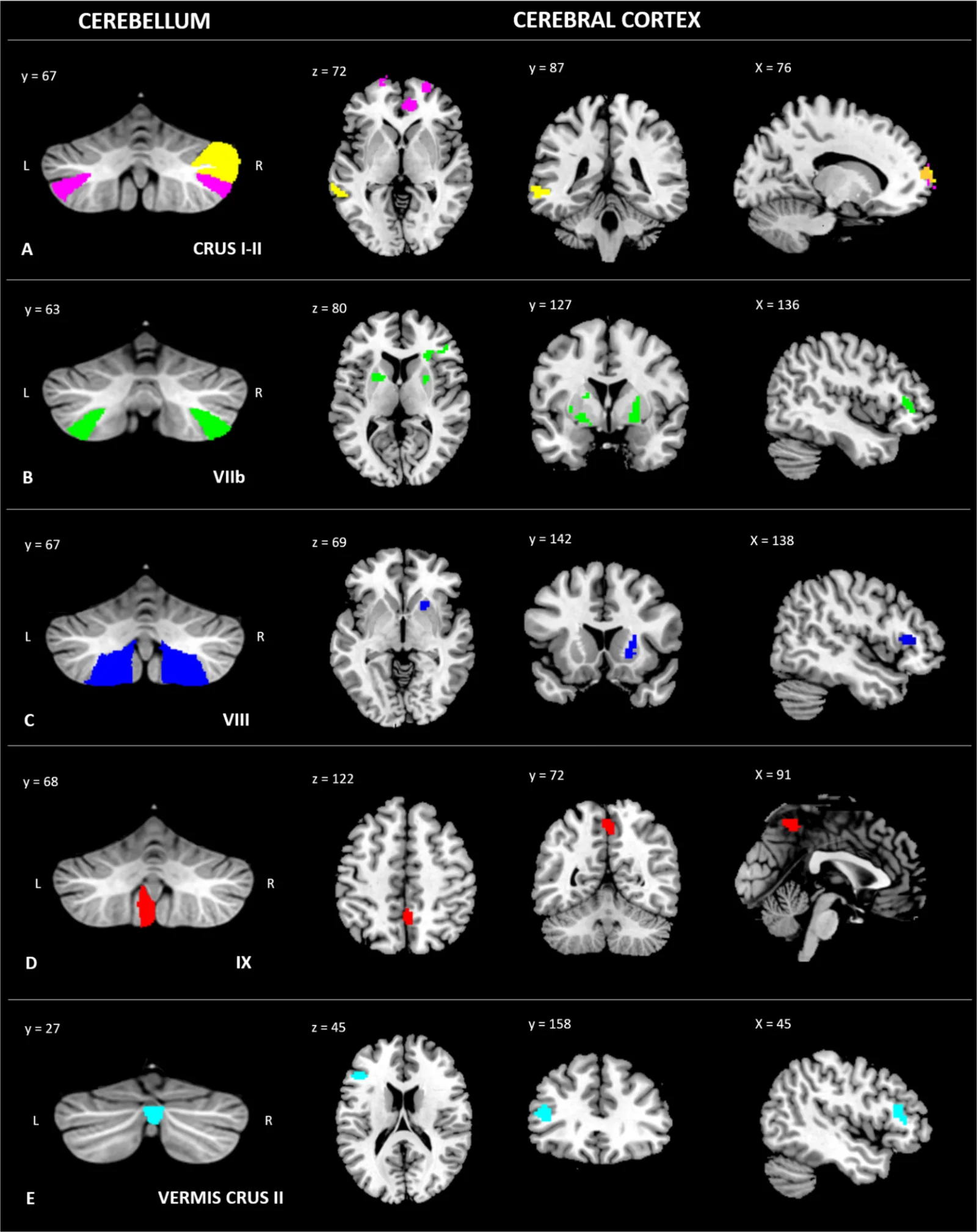
Cerebello-cerebral FC. Cerebellar regions of interest (ROI) and corresponding clusters of decreased FC in the cerebral cortex. Cerebellar ROIs are shown in montreal neurological institute (MNI) space using the automated anatomical labeling 3 (AAL3) atlas [66]: (**A**) cerebellar right crus I, in yellow, and bilateral crus II, in magenta; (**B**) ROIs of bilateral cerebellar VIIb, in green; (**C**) ROIs of bilateral cerebellar VIII, in blue; (**D**) ROI of left IX, in red; (**E**) ROI of cerebellar vermis Crus II, in cyan. Clusters of significantly decreased FC in the cerebral cortex of patients are shown in axial (z), coronal (y) and sagittal (x) slices in MNI space. Cluster-level FWE correction (*p* < 0.05). Detailed statistics and MNI coordinates corresponding to the maximum statistical value within each significant cluster are reported in Table 2. R = right, L = left

Compared to controls, CB patients exhibited several patterns of decreased FC between posterior and vermian cerebellar ROIs and regions of the cerebral cortex linked to social functions or prediction processing (Fig. 9; Table 2). The right Crus I showed decreased FC with the Superior Frontal Gyrus and the Middle Temporal Gyrus, while the right Crus II displayed decreased FC with both the Superior and the Middle Frontal Gyrus. The left Crus II showed reduced FC with the vmPFC, while no patterns of altered FC involved the left Crus I. The right lobule VIIb showed decreased FC with the right putamen and the left pallidum, while the right lobule VIII showed reduced FC with the right putamen alone. Both the left lobules VIIb and VIII exhibited decreased FC with the right Inferior Frontal Gyrus. Lastly, the left lobule IX showed a reduced FC with the left precuneus, and the vermian Crus II was less connected with the left inferior frontal gyrus. No pattern of increased FC was identified. Detailed statistics of seed-based analyses are reported in Table 2.

**Table 2:** Statistics of cerebellar ROIs and corresponding clusters of decreased FC in the cerebral cortex (CB < HS)

Cerebellar ROIs	Cluster size (NoV)	MNI Coordinates			Cluster peak Z-score	Brain regions
		x	y	z		
R Crus I	72	-62	-39	-5	3.99	L Middle Temporal Gyrus
	55	-17	68	14	4.57	L Superior Frontal Gyrus
R Crus II	80	-17	68	14	4.57	L Superior Frontal Gyrus
	60	-37	28	48	3.79	L Middle Frontal Gyrus
L Crus II	128	30	59	-11	4.25	R Frontal Middle Orbital (vmPFC)
	65	8	45	0	3.88	R Frontal Middle Orbital (vmPFC)
R VIIb	87	25	3	6	3.37	R Putamen
	94	-26	-8	-5	4.48	L Pallidum
L VIIb	84	56	31	20	4.13	R Inferior Frontal Gyrus
R VIII	137	25	17	12	3.62	R Putamen
L VIII	63	47	23	9	3.81	R Inferior Frontal Gyrus
L IX	63	-0	-50	54	4.29	L Precuneus
Vermian Crus II	57	-43	28	14	3.72	L Inferior Frontal Gyrus

The table reports MNI coordinates (x, y, z) in the Montreal Neurological Institute space and peak Z score of the peak voxels which exhibited the greatest statistical differences in a cluster. Only regions that survived after correction for multiple comparisons (FWE corrected *p* < 0.05) were reported. *NoV* number of voxels, *R* right, *L* left

### Exploratory Correlation with Cerebellar-Cerebral FC Estimates

**Fig. 10:**
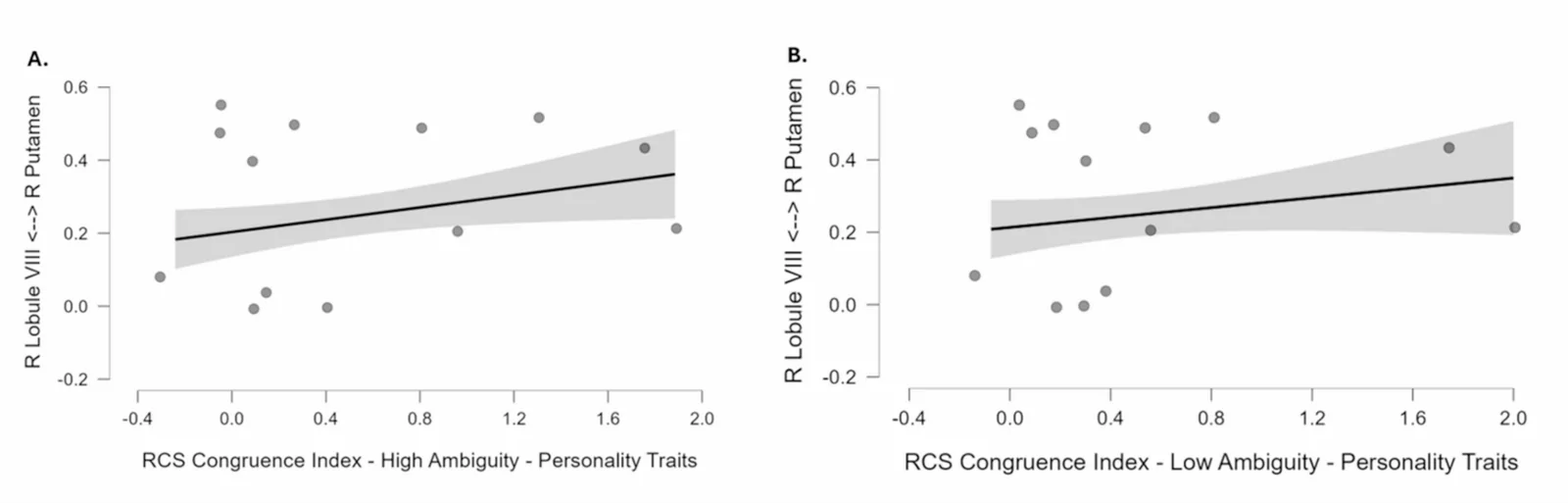
Scatterplots of significant correlations between FC values and CIs. Data scatterplots of significant correlation between higher values of decreased right cerebellar lobule VIII - right putamen FC and patients’ higher CIs in high (**A**) and low (**B**) ambiguity levels of personality traits prediction task

Among the exploratory brain–behaviour association, Spearman’s correlation analysis disclosed that higher values of decreased cerebellar-cerebral FC between the right cerebellar lobule VIII and the right putamen were significantly correlated with higher patients’ CIs in both high (*r* = 0.621, *p* = 0.027; Fig. 10A) and low (*r* = 0.593, *p* = 0.036; Fig. 10B) ambiguity levels of the personality traits prediction task. Both associations did not survive Benjamini–Hochberg correction across the full set of brain–behaviour correlations (see “Discussion” section). Partial correlations controlling for total motor severity were subsequently examined, further motivated by the knowledge that both lobule VIII and the putamen are involved in sensorimotor processing [72, 78]. The magnitude of the associations remained significant for both high (partial *r* = 0.629, *p* = 0.029) and low (partial *r* = 0.590, *p* = 0.043) ambiguity, indicating minimal attenuation after adjustment for clinical motor severity. Whole exploratory brain–behaviour correlations are reported in Table S9 in Supplementary Materials.

## Discussion

The present study investigated the role of cerebello-cortical networks in context-based social prediction by combining behavioural measures with resting-state FC analyses in individuals with cerebellar degeneration and matched healthy controls. Participants completed three context-based prediction tasks where contextual priors activate specific internal models setting expectations which might influence ambiguous targets’ classification across two social domains (emotion expression prediction and personality traits prediction) and a non-social control domain. These contextual expectations were followed by ambiguous target stimuli conveying social or non-social information (emotion expression, personality traits, or objects) that were either congruent or incongruent with the preceding contextual information. Overall, the findings support the hypothesis that cerebellar degeneration disrupts predictive mechanisms underlying complex social cognition and suggest that these impairments are associated with altered FC within cerebello-cerebral networks supporting social and predictive processing.

At the behavioural level, main effects of stimulus ambiguity and contextual congruency on accuracy were observed across all prediction tasks. Across groups, participants showed lower accuracy for more ambiguous stimuli and benefited from congruent contextual priors, confirming that both the perceptual legibility of stimuli and contextual facilitation jointly shape behavioural performance. A similar pattern emerged for RTs, although the congruency effect reached significance only in the personality traits prediction task.

Importantly, group differences were selective rather than generalized. Patients with cerebellar degeneration performed comparably to healthy controls in the emotion prediction task, suggesting that the use of contextual information to predict basic emotional expressions conveyed by immediately available facial cues remains largely preserved following adult-onset cerebellar degeneration. Although based on a different stimulus modality, this finding is consistent with previous evidence showing preserved attribution of basic emotions from contextual information presented through short stories in cerebellar patients [34].

By contrast, behavioural impairments emerged in the personality traits prediction task, as evidenced by a significant Group × Congruency interaction. Specifically, patients with cerebellar degeneration showed reduced accuracy selectively in incongruent trials. Within the patient group, performance was significantly poorer in incongruent than in congruent conditions, whereas between-group comparisons revealed that patients performed worse than healthy controls only when contextual information conflicted with the target stimulus. Notably, this effect was independent of stimulus ambiguity, indicating that the deficit cannot be explained by reduced perceptual processing of the target stimuli, but it rather reflects difficulty in flexibly integrating contextual information when prior expectations must be updated. This interpretation is further supported by the analysis of the CI, that represents a measure of the extent to which contextual priors influence behavioural responses. While no group differences emerged for the emotion prediction or non-social control tasks, cerebellar patients showed significantly higher CI values than healthy controls in the personality traits prediction task. This finding indicates a stronger reliance on contextual priors, suggesting that patients tended to maintain context-driven interpretations even when incoming information contradicted their initial expectations and should have triggered prediction-error updating. Together with the selective reduction in accuracy observed during incongruent personality traits trials, these results converge in indicating that cerebellar degeneration does not simply reduce performance but specifically impairs the flexible revision of internal models in response to conflicting social evidence. This difficulty appears to emerge particularly when social judgements require higher-order inferential processing, such as personality traits attribution from facial expressions, where integrating contextual information and revising previously established interpretations are essential for accurate social prediction. The selective impairment observed in the personality traits prediction task could be consistent with the different social demands imposed by the two social tasks. Personality traits attribution from facial expressions requires the construction of more abstract and stable representations of others by integrating contextual information with prior social knowledge, whereas emotion recognition of basic emotional expressions from faces relies more heavily on the processing of immediately available affective cues [79]. A similar distinction was proposed by Coricelli [80], who differentiated relatively automatic components of social cognition from higher-order inferential processes requiring the anticipation and evaluation of others’ mental states through comparison with previously acquired internal representations. Within this framework, personality traits attribution places greater demands on the integration of contextual information and, under incongruent conditions, requires the revision of previously established representations in light of conflicting evidence.

The present findings extend previous evidence obtained using context-based social prediction paradigms. Butti and colleagues [81] reported broader impairments in paediatric patients with congenital or early-acquired cerebellar damage, who showed reduced performance in both congruent and incongruent conditions in predictive tasks. The authors suggested that early cerebellar damage interferes with the development of temporally and spatially organized internal models linking contextual information to subsequent social events [81], in accordance with the sequence detection theory proposed by Leggio and colleagues [29, 82]. Importantly, evidence from studies involving adults further indicates that the cerebellum is involved in the construction and processing of socially meaningful action sequences. Indeed, patients with adult-onset cerebellar degeneration showed difficulties in reconstructing false-belief action sequences [48], while neuroimaging evidence in healthy adults demonstrated the involvement of posterior cerebellar regions in constructing both false- and true-belief social action sequences [35]. By contrast, the patients included in the present study developed cerebellar pathology during adulthood, when internal models supporting commonly experienced social situations are likely to have already been established before disease onset and clinical symptoms manifestation. Within the context-based prediction paradigm used here, we therefore hypothesize that the prime stimuli may activate previously acquired internal models, which remain available despite the subsequent development of cerebellar pathology but may be less efficiently updated when incoming information conflicts with the expectations they elicit. Accordingly, patients performed similarly to healthy controls in conditions relying on congruent contextual information and on relatively automatic components of social processing, such as emotion recognition conveyed by immediately available facial cues. In contrast, impairments emerged when social judgements required higher-order inferential processing and the revision of prior expectations in response to incongruent contextual information, as in the personality traits prediction task. Additionally, in line with our results, neuroimaging studies have consistently reported greater recruitment of posterior cerebellar regions during tasks involving personality traits inference and the prediction of socially meaningful action sequences, particularly when contextual expectations are violated [83–85].

Overall, within the predictive coding framework, the present findings suggest that cerebellar degeneration may particularly affect the updating of previously established internal models when incoming complex social information conflicts with contextual expectations, thereby reducing the flexibility with which existing internal models can be adapted to novel social information. Such a deficit is consistent with current models proposing that the cerebellum contributes to prediction-error detection and the continuous refinement of internal models required to optimize behaviour [24].

Importantly, the behavioural findings were paralleled by patterns of decreased FC within cerebellar-cerebral circuits in cerebellar patients compared to healthy controls, suggesting a disruption of large-scale networks involved in different features of cognitive and social functions and thus providing a potential neural substrate for the observed selective impairment in complex social prediction. Specifically, the FC dysfunction involving lobules Crus I and II, VIIb, VIII, IX, and the vermian Crus II mainly converge onto cortical nodes belonging to the DMN, salience network and executive-control systems, as well as basal ganglia implicated in adaptive updating [69, 86–88].

The decreased coupling between the right Crus I and the superior frontal gyrus and middle temporal gyrus, together with the reduced FC between bilateral Crus II and medial and lateral prefrontal areas and between the left Crus II and vmPFC, points to a disruption of the cerebellar-cerebral networks supporting higher-order social inferences [36]. The posterior cerebellar Crus I and Crus II are consistently and most strongly linked to the mentalizing networks, including the medial PFC, temporoparietal regions, and the temporal cortex [36, 41, 87, 89]. The middle temporal gyrus is a key region involved in processing socially relevant facial information, emotion recognition and semantic representations supporting social knowledge [90, 91]. Recent meta-analytic evidence further indicates a partial overlap between semantic and social mentalizing processes, with the left middle temporal gyrus among the regions recruited by both semantic and social mentalizing content [92, 93]. In parallel, the vmPFC has been consistently implicated in the representation of social knowledge, the integration of value-related information about others and the creation of abstract models that support predictions about other people’s traits and behaviour [94–97]. Consistently, a recent study investigating effective connectivity at rest in SCA2 through Dynamic Causal Modelling showed that, compared with healthy controls, individuals with SCA2 had reduced inhibitory effective connectivity from the left Crus II to the vmPFC and to the precuneus, indicating a weakened cerebellar modulatory influence on cortical regions. Conversely, inhibitory connectivity from the vmPFC to the left Crus II was stronger, suggesting enhanced cortical inhibition of cerebellar activity [41]. Accordingly, the decreased connectivity between the left Crus II and the vmPFC may compromise the reciprocal exchange between cerebellar prediction-error computations and cortical representations of social expectations, ultimately favouring the persistence of prior beliefs despite contradictory evidence. Also, reduced FC between the left lobule IX and the precuneus was observed in CB patients. This result is consistent with resting-state studies in healthy individuals, which identified lobule IX as a cerebellar representation of DMN, showing intrinsic functional coupling with cortical DMN regions, including the precuneus/posterior cingulate cortex [86, 87]. Recently, lobule IX has also been implicated in social predictive processing, being preferentially recruited during the prediction of social action sequences based on traits information and proposed to contribute to the motivational aspects of anticipating future social interactions [43, 83]. In this vein, disrupted communication between lobule IX and the precuneus in CB patients may further impair the adaptive revision of previously learned social representations on the basis of contradictory actual stimuli, which may impede the implementation of flexible models in the future.

Additionally, reduced connectivity between bilateral lobules VIIb–VIII, the vermian Crus II, and the inferior frontal gyrus may reflect a diminished capacity to use discrepant information to modify ongoing interpretations, pointing to dysfunction within networks supporting salience processing. Indeed, the inferior frontal gyrus has been implicated in response inhibition, attentional reorienting, and the detection of behaviourally relevant signals, thus supporting the interruption of ongoing processing and the flexible reconfiguration of behaviour when salient or unexpected events occur [98–100].

Overall, current literature provides evidence that the mentioned cerebellar-cerebral networks are thought to support the construction of internal models of others’ intentions, traits, and dispositions, allowing predictions about social behaviour across contexts [36, 41]. Within this sophisticated process, the cerebellum detects mismatches between expected or anticipated events and ongoing interactions, triggering error signals that facilitate adjustments of social behaviour. Cerebellar damage may impair these predictive and error-monitoring functions, disrupting cerebellar modulation of social brain networks. Notably, the observed patterns of reduced FC between the posterior cerebellum and regions of the DMN and salience network provide further evidence of large-scale cerebello-cortical network disruption in cerebellar diseases, consistent with previous reports [34, 41].

A particularly novel and relevant finding was the reduced FC between right cerebellar lobules VIIb–VIII and the putamen and pallidum. Recent meta-analytic evidence provides an important functional framework for interpreting this finding. A meta-analysis of Van Overwalle et al. [37] showed that both the posterior cerebellum and anterior basal ganglia, mainly the caudate and to a lesser degree the putamen, contribute to dynamic social mentalizing, indicating partly distinct functional profiles. Indeed, the cerebellum showed a broader functional specialization for social processing, being preferentially recruited not only by social information per se, but also when social information involved sequencing or inconsistency. In contrast, the basal ganglia did not exhibit a general preference for social information; rather, their involvement appeared more selective, emerging specifically when social processing required the representation of action sequences or the detection of inconsistent social information. According to the authors, this pattern suggested that basal ganglia involvement in dynamic social mentalizing may be particularly relevant when understanding others requires the integration of temporally structured behavioural information, mostly when observed behaviour is inconsistent with previously established social representations. One possible interpretation is that the enhanced recruitment of the basal ganglia under such conditions reflects additional motivational demands associated with initiating or adapting actions to deal with these inconsistencies, potentially accounting for their more selective involvement compared with the cerebellum. Meta-analytic connectivity analyses further revealed coactivation between the posterior cerebellum, basal ganglia, and neocortical mentalizing regions (i.e., mPFC, TPJ, posterior superior temporal sulcus, and precuneus), supporting a distributed cortico-subcortical network for dynamic social mentalizing. These findings are particularly relevant to the present results, given that behavioural alterations emerged specifically when incoming social information was incongruent with previously established contextual expectations.

In this context, the exploratory association between reduced right lobule VIII–putamen FC and increased CI in the personality traits prediction task may point to a relationship between cerebello-striatal connectivity and greater reliance on contextual information during social prediction. However, this association did not survive correction for multiple comparisons and, given the limited sample size, should be interpreted cautiously and requires independent replication.

Importantly, anatomical evidence demonstrates bidirectional subcortical pathways linking cerebellar and basal ganglia territories through the thalamus and subthalamic nucleus [88, 101, 102]. Together with recent evidence for the joint involvement and coactivation of the posterior cerebellum and basal ganglia during dynamic social mentalizing, particularly during social sequencing and inconsistency processing [37], this anatomical and functional evidence provides a plausible network framework for the cerebellar-basal ganglia FC alterations observed in the present study.

At a broader theoretical level, the involvement of cortical, cerebellar, and basal ganglia regions in these processes may also be considered in light of models proposing complementary contributions of these systems to learning and adaptive behaviour [103, 104]. Within this theoretical framework, the cerebral cortex has been associated with the extraction of statistical regularities and the construction of internal representations, the cerebellum with prediction-based refinement of internal models, and the basal ganglia with reinforcement-based mechanisms contributing to adaptive behavioural updating [103, 104]. Although these computational mechanisms were not directly investigated in the present study, they provide a broader theoretical context for considering the interaction among cortical, cerebellar, and basal ganglia systems. Indeed, rather than operating independently, these systems interact continuously, potentially allowing prediction-error-related information to shape behavioural updating through cerebello-striatal communication while cortical representations provide the contextual framework within which learning occurs [103, 104].

Overall, the widespread reduction in cerebellar FC may reflect impairment of an integrated learning architecture involving cortical, cerebellar and basal ganglia networks.

Notably, the behavioural alterations observed in the personality traits task, but not in basic emotion recognition (i.e., joy and anger), suggest that cerebellar degeneration may particularly affect higher-order predictive social cognition, which strongly engages posterior cerebellar regions. Indeed, unlike basic emotion recognition, personality traits attribution requires the integration of contextual information over time, the preservation of abstract representations of others’ characteristics, and the flexible updating of these internal models when new evidence contradicts prior expectations, functions that, as previously discussed, likely depend on the coordinated interaction of multiple cortical, cerebellar and striatal networks. In contrast, recognition of basic emotional expressions may rely on more strongly learned and evolutionarily conserved predictive models supported by distributed vermian-limbic networks [68].

Although the present findings provide novel insights into the neural mechanisms underlying social prediction in cerebellar neurodegeneration, some limitations must be acknowledged. First, because of the relatively small sample size, which reflects both the rarity of cerebellar neurodegenerative disorders and the stringent inclusion criteria that we adopted, the statistical power to detect additional significant correlations may have been limited. Also, the clinical heterogeneity of the patient group may have introduced additional variability. Therefore, future studies should aim to replicate these findings in larger cohorts and in more homogeneous clinical populations. A potential limitation of the study is that ambiguity was operationalized differently across tasks. However, this difference was inherent to the distinct nature of the stimuli and does not substantially affect the interpretation of the findings, as ambiguity was manipulated to generate different levels of uncertainty within each task, rather than to provide quantitatively equivalent levels across tasks. Moreover, each task was analysed separately, and no direct comparison of ambiguity effects across tasks was intended. Another limitation concerns the exploratory nature of brain-behaviour correlations.

Moreover, the seed-based resting-state FC approach allows identifying statistical dependencies between brain regions but does not provide information about the directionality or causal influence of these interactions. Future investigations could therefore benefit from applying effective connectivity analysis, such as Dynamic Causal Modelling, to characterize the directional interactions within cerebello-cortical and cerebello-striatal networks. Also, this advanced approach could more likely unfold the causal inferences regarding the computational mechanisms underlying altered predictive processing in cerebellar neurodegeneration, which cannot be elucidated by the present findings. Overall, further studies in larger and clinically homogeneous cohorts, integrating advanced connectivity analyses, will be essential to validate and extend these findings and to clarify how alterations of cerebello-cortical and cerebello-striatal networks contribute to deficits in predictive social cognition in cerebellar neurodegenerative diseases.

## Conclusion

The present findings evidenced that patients with cerebellar neurodegenerative diseases showed reduced performance and a stronger reliance on contextual priors in the personality traits prediction task. Conversely, this pattern was not observed in the emotion prediction task nor in the non-social control task. These results suggest that cerebellar degeneration compromises the flexible updating of social internal models when contextual information conflicts with incoming evidence. This impairment appears to emerge selectively in higher-order social inferences, which rely on abstract representations and require greater contextual integration and active model updating to support accurate social prediction.

The behavioural findings were paralleled by widespread reduction in FC between posterior and vermian cerebellar lobules (Crus I, Crus II, VIIb, VIII, IX, Vermian Crus II) and cortical and subcortical nodes supporting social cognition, salience processing and behavioural updating, including regions of the DMN, the inferior frontal gyrus, and the basal ganglia. Within this large-scale network disruption, the exploratory association between reduced right lobule VIII–putamen FC and greater contextual dependence during personality traits prediction suggests that altered cerebello-striatal communication may be related to reduced flexibility in responding to social information that conflicts with previously established contextual expectations. Although preliminary and exploratory, these findings support a network-level account of impaired social prediction in cerebellar degeneration, with higher-order inferences being particularly vulnerable when prior representations need to be updated in light of conflicting evidence. 

## Supplementary Information

Below is the link to the electronic supplementary material.

Supplementary Material 1

## Data Availability

The datasets used and/or analysed during the current study are available from the corresponding author on reasonable request.
