# Supplementary material for: Large-Scale Cerebellar-Cerebral Network Alterations Underlying Impaired Social Predictive Processing in Cerebellar Neurodegeneration
Source: Cerebellum. 2026 Oct 6;25(5):150. doi: 10.1007/s12311-026-02095-y (PMC13642411; doi:10.1007/s12311-026-02095-y)
Supplement: Supplementary file 1 [file 12311_2026_2095_MOESM1_ESM.docx]

**Figure S1.** T1-weighted images of each patient normalized to the Spatially Unbiased atlas template of the cerebellum and brainstem (SUIT, presented in the left lower panel; [1]) are displayed in representative sections, illustrating the range of cerebellar atrophy across subjects.

**Table S1.** Demographic and clinical characteristics of CB patients and HS for each task and measure after outliers’ data cleaning.

| **Task** | **Measures** | **Group** | **N** | **Age** | **Statistics** | **Education** | **Statistics** | **M/F** | **Statistics** |
| --- | --- | --- | --- | --- | --- | --- | --- | --- | --- |
| Emotion Expression Task | Accuracy | CB | 12 | 48.83  (13.18) | *p =.584*  *t =-.553* | 15.25  (2.7) | *p =.899*  *t =-.128* | 8/14 | *p =.86*  *χ² =.003* |
|  |  | HS | 22 | 50.55 (4.74) |  | 15.36  (2.34) |  | 4/8 |  |
|  | RTs | CB | 13 | 49.54  (12.88) | *p =.786*  *t =-.273* | 15.08  (2.66) | *p =.711*  *t =-.373* | 5/8 | *p =.825*  *χ² =.049* |
|  |  | HS | 23 | 50.35  (4.72) |  | 15.39  (2.29) |  | 8/15 |  |
|  | Congruency Index | CB | 13 | 49.54 (12.88) | *p =.711*  *t =-.374* | 15.08  (2.66) | *p =.678*  *t =-.419* | 5/8 | *p =.614*  *χ² =.254* |
|  |  | HS | 20 | 50.7  (4.4) |  | 15.54  (2.39) |  | 6/14 |  |
| Personality Traits Task | Accuracy | CB | 13 | 49.54  (12.88) | *p =.939*  *t =-.077* | 15.08  (2.66) | *p =.622*  *t =-.498* | 5/8 | *p =.901*  *χ² =.015* |
|  |  | HS | 22 | 49.77  (4.93) |  | 15.5  (2.28) |  | 8/14 |  |
|  | RTs | CB | 12 | 50.67 (12.76) | *p =.874*  *t =.16* | 14.83  (2.62) | *p =.294*  *t =-1.065* | 5/7 | *p =.739*  *χ² =.111* |
|  |  | HS | 25 | 50.2  (5.11) |  | 15.72  (2.25) |  | 9/16 |  |
|  | Congruency Index | CB | 13 | 49.54 (12.88) | *p =.78*  *t =-.282* | 15.08  (2.66) | *p =.455*  *t =-.756* | 5/8 | *p =.954*  *χ² =.003* |
|  |  | HS | 24 | 50.83  (5.14) |  | 15.71  (2.29) |  | 9/15 |  |
| Non-social Control Task | Accuracy | CB | 13 | 49.54  (12.88) | *p =.842*  *t =-.200* | 15.08  (2.66) | *p =.592*  *t =-.540* | 5/8 | *p =.927*  *χ² =.008* |
|  |  | HS | 25 | 50.12  (5.03) |  | 15.52  (2.26) |  | 10/15 |  |
|  | RTs | CB | 13 | 49.54  (12.88) | *p =.77*  *t =-.295* | 15.08  (2.66) | *p =.533*  *t =-.629* | 5/8 | *p =.881*  *χ² =.011* |
|  |  | HS | 25 | 50.4  (5.15) |  | 15.6  (2.31) |  | 9/16 |  |
|  | Congruency Index | CB | 12 | 49.42  (13.44) | *p =.757*  *t =-.312* | 15.5  (2.28) | *p =.885*  *t =-.146* | 4/8 | *p =.761*  *χ² =.093* |
|  |  | HS | 26 | 50.35  (5.06) |  | 15.62  (2.26) |  | 10/16 |  |

Data are reported as means and standard deviations for age and education. N = number of participants; F = female; M = male; RTs = reaction times.

**Table S2.** Descriptive statistics of the factors’ levels of the context-based prediction tasks expressed as mean and standard error of both accuracy and RTs. Values reflect the interaction effects among within subject factors.

|  |  | **Factors’ levels** | | | |
| --- | --- | --- | --- | --- | --- |
| Task | Measures | Congruence | | Ambiguity | |
|  |  | Congruent | Incongruent | More ambiguous | Less ambiguous |
| Emotion Expression | Accuracy | 0.785 (0.012) | 0.684  (0.019) | 0.676  (0.014) | 0.793  (0.017) |
|  | RTs | 1150.53  (56.6) | 1207.49  (58.8) | 1234.86  (62.46) | 1123.16  (52.035) |
| Personality Traits | Accuracy | 0.874  (0.013) | 0.743  (0.028) | 0.743  (0.022) | 0.874  (0.021) |
|  | RTs | 1048.29  (48.006) | 1139.4  (55.495) | 1149.84  (52.547) | 1037.85  (50.931) |
| Non-social prediction | Accuracy | 0.786  (0.017) | 0.743  (0.017) | 0.756  (0.018) | 0.773  (0.017) |
|  | RTs | 1183.59  (61.210) | 1182.47  (69.724) | 1173.05  (64.760) | 1193.01  (66.420) |

**Table S3.** Descriptive statistics of the context-based prediction tasks in each combination defined by the group and the congruence in each task, reported as mean and standard error of both the Accuracy and RTs. Values reflect the Congruence x Group interaction effects.

|  | | **Congruence x Group** | | | | | |  |
| --- | --- | --- | --- | --- | --- | --- | --- | --- |
| Groups | Measures | Emotion expression | | Personality traits | | Non-social prediction | |  |
|  |  | Congruent | Incongruent | Congruent | Incongruent | Congruent | Incongruent | |
| CB | Accuracy | 0.744  (0.021) | 0.648  (0.029) | 0.881  (0.019) | 0.621  (0.060) | 0.713  (0.040) | 0.641  (0.034) | |
|  | RTs | 1463.13  (122.767) | 1502.76  (130.519) | 1342.37  (97.888) | 1489.2  (123.111) | 1654..88  (124.827) | 1684.22  (155.173) | |
| HS | Accuracy | 0.807  (0.014) | 0.703  (0.025) | 0.869  (0.017) | 0.815  (0.022) | 0.824  (0.014) | 0.797  (0.016) | |
|  | RTs | 973.843  (35.725) | 1040.6  (38.579) | 907.134  (40.848) | 971.5  (40.020) | 938.528  (31.625) | 921.559  (29.345) | |

**Table S4.** Descriptive statistics of Emotion expression prediction tasks in each combination defined by the congruence and the ambiguity, reported as mean accuracies and standard error per group and in the entire sample. Values reflect the Congruence x Ambiguity interaction effects.

|  |  | **Emotion expression task - Congruence x Ambiguity** | | | |
| --- | --- | --- | --- | --- | --- |
| Groups | Measures | Congruent –  High Ambiguity | Congruent - Low Ambiguity | Incongruent - High Ambiguity | Incongruent - Low Ambiguity |
| CB | Accuracy | 0.689  (0.031) | 0.597  (0.018) | 0,597  (0,027) | 0.7  (0.048) |
|  | RTs | 1556.507  (196.318) | 1369.75  (151.01) | 1562.44  (204.675) | 1443.08  (168.905) |
| HS | Accuracy | 0.748  (0.015) | 0.865  (0.016) | 0.639  (0.030) | 0.767  (0.037) |
|  | RTs | 1021.106  (55.354) | 926.579  (44.205) | 1081.66  (54.710) | 999.533  (54.243) |
| All Participants | Accuracy | 0.727  (0.015) | 0.842  (0.013) | 0.625  (0.022) | 0.743  (0.029) |
|  | RTs | 1214.445  (88.835) | 1086.61  (70.019) | 1255.28  (88.951) | 1159.7  (77.500) |

**Table S5.** Descriptive statistics of Personality traits prediction tasks in each combination defined by the congruence and the ambiguity, reported as mean accuracies and standard error per group and in the entire sample. Values reflect the Congruence x Ambiguity interaction effects.

|  |  | **Personality traits - Congruence x Ambiguity** | | | |
| --- | --- | --- | --- | --- | --- |
| Groups | Measures | Congruent –  High Ambiguity | Congruent –Low Ambiguity | Incongruent – High Ambiguity | Incongruent –  Low Ambiguity |
| CB | Accuracy | 0.823  (0.027) | 0.940  (0.014) | 0.553  (0.083) | 0.688  (0.087) |
|  | RTs | 1403.901  (138.528) | 1280.841  (142.099) | 1544.252  (175.562) | 1434.143  (178.908) |
| HS | Accuracy | 0.811  (0.026) | 0.928  (0.015) | 0.739  (0.028) | 0.892  (0.025) |
|  | RTs | 955.092  (61.379) | 859.176  (53.426) | 1033.323  (59.514) | 909.676  (51.765) |
| All Participants | Accuracy | 0.811  (0.019) | 0.932  (0.011) | 0.670  (0.038) | 0.816  (0.039) |
|  | RTs | 1100.65  (69.461) | 995.932  (66.099) | 1199.03  (78.987) | 1079.774  (77.803) |

**Table S6.** Descriptive statistics of Non-social control prediction tasks in each combination defined by the congruence and the ambiguity, reported as mean accuracies and standard error per group and in the entire sample. Values reflect the Congruence x Ambiguity interaction effects.

|  |  | **Non-social control** **Task - Congruence x Ambiguity** | | | |
| --- | --- | --- | --- | --- | --- |
| Groups | Measures | Congruent –  High Ambiguity | Congruent - Low Ambiguity | Incongruent - High Ambiguity | Incongruent - Low Ambiguity |
| CB | Accuracy | 0.705  (0.055) | 0.721  (0.062) | 0.615  (0.048) | 0.667  (0.048) |
|  | RTs | 1596.857  (165.923) | 1712.908  (191.918) | 1670.649  (240.816) | 1697.792  (205.679) |
| HS | Accuracy | 0.826  (0.019) | 0.822  (0.021) | 0.786  (0,023) | 0.808  (0.022) |
|  | RTs | 951.086  (45.015) | 925.968  (45.216) | 915.894  (38.923) | 927.223  (44.708) |
| All Participants | Accuracy | 0.784  (0.024) | 0.787  (0.026) | 0.727  (0.026) | 0.759  (0.024) |
|  | RTs | 1172.008  (80.349) | 1195.185  (93.414) | 1174.100  (102.694) | 1190.839  (95.700) |

**Table S7.** Descriptive statistics of the context-based prediction tasks in each combination defined by the group and the ambiguity, reported as mean and standard error of the Congruency Indices. Values reflect Group x Ambiguity interaction effects.

| **Group x Ambiguity** | | | | | | | |  |  |
| --- | --- | --- | --- | --- | --- | --- | --- | --- | --- |
| Groups | Emotion expression | | Personality traits | | Non-social prediction | | |  |  |
|  | High Ambiguity | Low Ambiguity | High Ambiguity | Low Ambiguity | High Ambiguity | Low Ambiguity | | |  |
| CB | 0.121  (0.068) | 0.185  (0.086) | 0.518  (0.195) | 0.491  (0.178) | 0.172  (0.082) | | 0.113  (0.054) | | |
| HS | 0.163  (0.063 | 0.179  (0.068 | 0.209  (0.072) | 0.110  (0.065) | 0.078  (0.036) | | 0.006  (0.028) | | |

**Table S8.** Descriptive statistics of the context-based prediction tasks defined by group, reported as mean and standard error of the Congruency Indices. Values reflect the main effects of Group.

| **Group** | | | |  |
| --- | --- | --- | --- | --- |
| Groups | Emotion expression | Personality traits | Non-social prediction |  |
| CB | 0.153  (0.054) | 0.504  (0.129) | 0.143  (0.05) | |
| HS | 0.171  (0.046) | 0.159  (0.048) | 0.041  (0.023) | |

**Table S9.** Exploratory brain–behaviour correlations. Exploratory Spearman correlations between individual FC estimates extracted from clusters showing reduced FC in CB patients and the Congruency Index (CI) of the personality prediction task. *p* values were adjusted for multiple comparisons using the Benjamini–Hochberg procedure. Nominally significant (uncorrected) correlations are shown in bold. Significant difference = *p<0.05.

| Reduced FC patterns | Congruency Indices | Spearman's ρ | p | Adjusted p (BH) |
| --- | --- | --- | --- | --- |
| R Crus I – L Superior Frontal Gyrus | CI – High Ambiguity | +0.159 | .604 | .982 |
|  | CI – Low Ambiguity | +0.220 | .470 | .940 |
| R Crus I – L Middle Temporal Gyrus | CI – High Ambiguity | +0.242 | .426 | .923 |
|  | CI – Low Ambiguity | +0.297 | .325 | .789 |
| R Crus II – L Superior Frontal Gyrus | CI – High Ambiguity | −0.082 | .792 | 1.000 |
|  | CI – Low Ambiguity | −0.115 | .710 | 1.000 |
| R Crus II – L Middle Frontal Gyrus | CI – High Ambiguity | −0.077 | .807 | 1.000 |
|  | CI – Low Ambiguity | −0.192 | .529 | .982 |
| L Crus II – R Middle Orbital Gyrus (PFCventmed) | CI – High Ambiguity | +0.000 | 1.000 | 1.000 |
|  | CI – Low Ambiguity | +0.011 | .978 | 1.000 |
| L Crus II – R Middle Orbital Gyrus (PFCventmed) (Cluster 2) | CI – High Ambiguity | −0.071 | .821 | 1.000 |
|  | CI – Low Ambiguity | −0.104 | .737 | 1.000 |
| R VIIb – L Putamen | CI – High Ambiguity | +0.396 | .182 | .614 |
|  | CI – Low Ambiguity | +0.291 | .334 | .789 |
| R VIIb – R Pallidum | CI – High Ambiguity | +0.495 | .089 | .504 |
|  | CI – Low Ambiguity | +0.390 | .189 | .614 |
| L VIIb – R Inferior Frontal Gyrus | CI – High Ambiguity | +0.159 | .604 | .982 |
|  | CI – Low Ambiguity | −0.060 | .849 | 1.000 |
| R VIII – R Putamen | **CI – High Ambiguity** | **+0.621** | **.027*** | **.468** |
|  | **CI – Low Ambiguity** | **+0.593** | **.036*** | **.468** |
| L VIII – R Inferior Frontal Gyrus | CI – High Ambiguity | +0.484 | .097 | .504 |
|  | CI – Low Ambiguity | +0.302 | .315 | .789 |
| L IX – L Precuneus | CI – High Ambiguity | +0.401 | .176 | .614 |
|  | CI – Low Ambiguity | +0.489 | .093 | .504 |
| Vermian Crus II – L Inferior Frontal Gyrus | CI – High Ambiguity | −0.022 | .949 | 1.000 |
|  | CI – Low Ambiguity | −0.011 | .978 | 1.000 |

**fMRI methods supplementary**

*fMRIPrep pipeline*

Results included in this manuscript come from preprocessing performed using *fMRIPrep* 25.1.4 (@fmriprep1; @fmriprep2; RRID:SCR_016216), which is based on *Nipype* 1.10.0 (@nipype1; @nipype2; RRID:SCR_002502).

Preprocessing of B_0_ inhomogeneity mappings: A total of 1 fieldmaps were found available within the input BIDS structure for this particular subject. A *B_0_*-nonuniformity map (or *fieldmap*) was estimated based on two (or more) echo-planar imaging (EPI) references with `topup` (@topup; FSL None).

Anatomical data preprocessing: A total of 1 T1-weighted (T1w) images were found within the input BIDS dataset. The T1w image was corrected for intensity/non-uniformity (INU) with `N4BiasFieldCorrection` [@n4], distributed with ANTs 2.6.2 [@ants, RRID:SCR_004757], and used as T1w-reference throughout the workflow. The T1w-reference was then skull-stripped with a *Nipype* implementation of the `antsBrainExtraction. sh` workflow (from ANTs), using OASIS30ANTsas target template. Brain tissue segmentation of cerebrospinal fluid (CSF), white-matter (WM) and gray-matter (GM) was performed on the brain-extracted T1w using `fast` [FSL (version unknown), RRID:SCR_002823, @fsl_fast]. Brain surfaces were reconstructed using `recon-all` [FreeSurfer 7.3.2, RRID:SCR_001847, @fs_reconall], and the brain mask estimated previously was refined with a custom variation of the method to reconcile ANTs-derived and FreeSurfer-derived segmentations of the cortical GM of Mindboggle [RRID:SCR_002438, @mindboggle]. A FLAIR image was used to improve pial surface refinement. Volume-based spatial normalization to one standard space (MNI152NLin2009cAsym) was performed through nonlinear registration with `antsRegistration` (ANTs 2.6.2), using brain-extracted versions of both T1w reference and the T1w template. The following template was selected for spatial normalization and accessed with *TemplateFlow* [24.2.2, @templateflow]:*ICBM 152 Nonlinear Asymmetrical template version 2009c* [@mni152nlin2009casym, RRID:SCR_008796; TemplateFlow ID: MNI152NLin2009cAsym].

Functional data preprocessing: For each of the 1 BOLD runs found per subject (across all tasks and sessions), the following preprocessing was performed. First, a reference volume was generated from the shortest echo of the BOLD run, using a custom methodology of *fMRIPrep*, for use in head motion correction. Head-motion parameters with respect to the BOLD reference (transformation matrices, and six corresponding rotation and translation parameters) are estimated before any spatiotemporal filtering using `mcflirt` [FSL, @mcflirt]. The estimated *fieldmap* was then aligned with rigid-registration to the target EPI (echo-planar imaging) reference run. The field coefficients were mapped on to the reference EPI using the transform. The BOLD reference was then co-registered to the T1w reference using `bbregister` (FreeSurfer) which implements boundary-based registration [@bbr]. Co-registration was configured with six degrees of freedom. Several confounding time-series were calculated based on the*preprocessed BOLD*: framewise displacement (FD), DVARS and three region-wise global signals. FD was computed using two formulations following Power (absolute sum of relative motions, @power_fd_dvars) and Jenkinson (relative root mean square displacement between affines, @mcflirt). FD and DVARS are calculated for each functional run, both using their implementations in *Nipype* [following the definitions by @power_fd_dvars]. The three global signals are extracted within the CSF, the WM, and the whole-brain masks. Additionally, a set of physiological regressors were extracted to allow for component-based noise correction [*CompCor*, @compcor]. Principal components are estimated after high-pass filtering the*preprocessed BOLD* time-series (using a discrete cosine filter with128s cut-off) for the two *CompCor* variants: temporal (tCompCor) and anatomical (aCompCor). tCompCor components are then calculated from the top 2% variable voxels within the brain mask. For aCompCor, three probabilistic masks (CSF, WM and combined CSF+WM) are generated in anatomical space. The implementation differs from that of Behzadi et al. in that instead of eroding the masks by 2 pixels on BOLD space, a mask of pixels that likely contain a volume fraction of GM is subtracted from the aCompCor masks. This mask is obtained by dilating a GM mask extracted from the FreeSurfer's *aseg* segmentation, and it ensures components are not extracted from voxels containing a minimal fraction of GM. Finally, these masks are resampled into BOLD space and binarized by thresholding at 0.99 (as in the original implementation). Components are also calculated separately within the WM and CSF masks. For each CompCor decomposition, the *k* components with the largest singular values are retained, such that the retained components' time series are sufficient to explain 50 percent of variance across the nuisance mask (CSF, WM, combined, or temporal). The remaining components are dropped from consideration. The head-motion estimates calculated in the correction step were also placed within the corresponding confounds file. The confound time series derived from head motion estimates and global signals were expanded with the inclusion of temporal derivatives and quadratic terms for each [@confounds_satterthwaite_2013]. Frames that exceeded a threshold of 0.5 mm FD or 1.5 standardized DVARS were annotated as motion outliers. Additional nuisance time series are calculated by means of principal components analysis of the signal found within a thin band (*crown*) of voxels around the edge of the brain, as proposed by [@patriat_improved_2017]. All resamplings can be performed with *a single interpolation step* by composing all the pertinent transformations (i. e. head-motiontransform matrices, susceptibility distortion correction when available, and co-registrations to anatomical and output spaces). Gridded (volumetric) resamplings were performed using `nitransforms`, configured with cubic B-spline interpolation.

Many internal operations of *fMRIPrep* use*Nilearn* 0.11.1 [@nilearn, RRID:SCR_001362], mostly within the functional processing workflow. For more details of the pipeline, see [the section corresponding to workflows in *fMRIPrep*'s documentation] (https://fmriprep. readthedocs. io/en/latest/workflows. html "FMRIPrep's documentation").

\### Copyright Waiver. The above boilerplate text was automatically generated by fMRIPrep with the express intention that users should copy and paste this text into their manuscripts *unchanged*. It is released under the [CC0] (https://creativecommons. org/publicdomain/zero/1. 0/) license.

*Tedana pipeline*

This is the tedana_orig tree [2], which is very similar to the criteria of the MEICA v2.5 decision tree [3]. For a description of the decision tree steps, with the rationale for each step, see Olafsson and colleagues [4]. TE-dependence analysis was performed on input data using the tedana workflow [5]. An initial mask was generated from the first echo using nilearn's compute_epi_mask function. An adaptive mask was then generated using the dropout method(s), in which each voxel's value reflects the number of echoes with 'good' data. An adaptive mask was then generated using the dropout method(s), in which each voxel's value reflects the number of echoes with 'good' data. A two-stage masking procedure was applied, in which a liberal mask (including voxels with good data in at least the first echo) was used for optimal combination, T2*/S0 estimation, and denoising, while a more conservative mask (restricted to voxels with good data in at least the first three echoes) was used for the component classification procedure. A monoexponential model was fit to the data at each voxel using nonlinear model fitting in order to estimate T2* and S0 maps, using T2*/S0 estimates from a log-linear fit as initial values. For each voxel, the value from the adaptive mask was used to determine which echoes would be used to estimate T2* and S0. In cases of model fit failure, T2*/S0 estimates from the log-linear fit were retained instead. Multi-echo data were then optimally combined using the T2* combination method [6]. Principal component analysis based on the PCA component estimation with a Moving Average (stationary Gaussian) process [7] was applied to the optimally combined data for dimensionality reduction.

The following metrics were calculated: kappa, rho, countnoise, countsigFT2, countsigFS0, dice_FT2, dice_FS0, signal-noise_t, variance explained, normalized variance explained, d_table_score. Kappa (kappa) and Rho (rho) were calculated as measures of TE-dependence and TE-independence, respectively. A t-test was performed between the distributions of T2*-model F-statistics associated with clusters (i.e., signal) and non-cluster voxels (i.e., noise) to generate a t-statistic (metric signal-noise_z) and p-value (metric signal-noise_p) measuring relative association of the component to signal over noise. The number of significant voxels not from clusters was calculated for each component. Independent component analysis was then used to decompose the dimensionally reduced dataset. RobustICA package was used for ICA decomposition [8]. The following metrics were calculated: countnoise, countsigFS0, countsigFT2, d_table_score, dice_FS0, dice_FT2, kappa, normalized variance explained, rho, signal-noise_t, variance explained. Kappa (kappa) and Rho (rho) were calculated as measures of TE-dependence and TE-independence, respectively. A t-test was performed between the distributions of T2*-model F-statistics associated with clusters (i.e., signal) and non-cluster voxels (i.e., noise) to generate a t-statistic (metric signal-noise_z) and p-value (metric signal-noise_p) measuring relative association of the component to signal over noise. The number of significant voxels not from clusters was calculated for each component.

Next, component selection was performed to identify BOLD (TE-dependent) and non-BOLD (TE-independent) components using a decision tree. Minimum image regression was then applied to the data in order to remove spatially diffuse noise [3].

This workflow used numpy [9], scipy [10], pandas [11,12], scikit-learn [13], nilearn, bokeh [14], matplotlib [15], and nibabel [16]. This workflow also used the Dice similarity index [17,18].

**Bibliography**

1. Diedrichsen J, Balsters JH, Flavell J, Cussans E, Ramnani N. A probabilistic MR atlas of the human cerebellum. Neuroimage [Internet]. Elsevier B.V.; 2009;46:39–46. https://doi.org/10.1016/j.neuroimage.2009.01.045

2. tedana community. Component selection decision trees in tedana. figshare. https://doi.org/10.6084/m9.figshare.25251433.v2. 2024.

3. Kundu P, Brenowitz ND, Voon V, Worbe Y, Vértes PE, Inati SJ, et al. Integrated strategy for improving functional connectivity mapping using multiecho fMRI. Proc Natl Acad Sci U S A. 2013;110:16187–92. https://doi.org/10.1073/pnas.1301725110

4. Olafsson V, Kundu P, Wong EC, Bandettini PA, Liu TT. Enhanced identification of BOLD-like components with multi-echo simultaneous multi-slice (MESMS) fMRI and multi-echo ICA. Neuroimage. 2015;112:43–51. https://doi.org/10.1016/j.neuroimage.2015.02.052

5. DuPre E, Salo T, Ahmed Z, Bandettini P, Bottenhorn K, Caballero-Gaudes C, et al. TE-dependent analysis of multi-echo fMRI with tedana. J Open Source Softw. The Open Journal; 2021;6:3669. https://doi.org/10.21105/joss.03669

6. Posse S, Wiese S, Gembris D, Mathiak K, Kessler C, Grosse-Ruyken ML, et al. Enhancement of BOLD-contrast sensitivity by single-shot multi-echo functional MR imaging. Magn Reson Med. John Wiley and Sons Inc.; 1999;42:87–97. https://doi.org/10.1002/(SICI)1522-2594(199907)42:1<87::AID-MRM13>3.0.CO;2-O

7. Li Y, Adalı T, Calhoun VD. Estimating the number of independent components for functional magnetic resonance imaging data. Hum Brain Mapp. 2007;28:1251–66. https://doi.org/10.1002/hbm.20359

8. Anglada-Girotto M, Miravet-Verde S, Serrano L, Head SA. robustica: customizable robust independent component analysis. BMC Bioinformatics. 2022;23:519. https://doi.org/10.1186/s12859-022-05043-9

9. Van Der Walt S, Colbert SC, Varoquaux G. The NumPy array: a structure for efficient numerical computation. 2011; https://doi.org/10.1109/MCSE.2011.37

10. Virtanen P, Gommers R, Oliphant TE, Haberland M, Reddy T, Cournapeau D, et al. SciPy 1.0: fundamental algorithms for scientific computing in Python. Nat Methods. 2020;17:261–72. https://doi.org/10.1038/s41592-019-0686-2

11. McKinney W. Data Structures for Statistical Computing in Python. 2010. p. 56–61. https://doi.org/10.25080/Majora-92bf1922-00a

12. The pandas development team. pandas-dev/pandas [Computer software]. Zenodo. 2020.

13. Pedregosa FABIANPEDREGOSA F, Michel V, Grisel OLIVIERGRISEL O, Blondel M, Prettenhofer P, Weiss R, et al. Scikit-learn: Machine Learning in Python Gaël Varoquaux Bertrand Thirion Vincent Dubourg Alexandre Passos PEDREGOSA, VAROQUAUX, GRAMFORT ET AL. Matthieu Perrot [Internet]. Journal of Machine Learning Research. 2011. http://scikit-learn.sourceforge.net.

14. Bokeh Development Team. Bokeh: Python library for interactive visualization [Computer software]. . 2018.

15. Hunter JD. Matplotlib: A 2D Graphics Environment. Comput Sci Eng. 2007;9:90–5. https://doi.org/10.1109/MCSE.2007.55

16. Brett M, Markiewicz CJ, Hanke M, Côté M-A, Cipollini B, McCarthy P, et al. nipy/nibabel: 2.4.1 (Version 2.4.1) [Computer software]. Zenodo. 2019.

17. Dice LR. Measures of the Amount of Ecologic Association Between Species. Ecology. 1945;26:297–302. https://doi.org/10.2307/1932409

18. Sorensen T. A Method of Establishing Groups of Equal Amplitude in Plant Sociology Based on Similarity of Species and Its Application to Analyses of the Vegetation on Danish Commons. . Biologiske skrifter; 1948.
